# Exogenously delivered iPSCs disrupt the natural repair response of endogenous MPCs after bone injury

**DOI:** 10.1038/s41598-023-36609-z

**Published:** 2023-06-09

**Authors:** Leah Ferrie, Priyatha Premnath, Alexandra Olsen, Leila Larijani, Bryce A. Besler, Derrick E. Rancourt, Neil A. Duncan, T. Michael Underhill, Roman J. Krawetz

**Affiliations:** 1 McCaig Institute for Bone and Joint Health, University of Calgary, Calgary, AB Canada; 2 Biomedical Engineering Graduate Program, University of Calgary, Calgary, AB Canada; 3 College of Engineering and Applied Science, University of Wisconsin Milwaukee, Milwaukee, WI USA; 4 Department of Biochemistry and Molecular Biology, Cumming School of Medicine, University of Calgary, Calgary, AB Canada; 5 Department of Civil Engineering, Schulich School of Engineering, University of Calgary, Calgary, AB Canada; 6 Department of Cellular and Physiological Sciences, University of British Columbia, Vancouver, BC Canada; 7 Department of Cell Biology and Anatomy, Cumming School of Medicine, University of Calgary, Calgary, AB Canada

**Keywords:** Musculoskeletal system, Bone, Osteoporosis, Pluripotent stem cells

## Abstract

Promoting bone healing including fracture non-unions are promising targets for bone tissue engineering due to the limited success of current clinical treatment methods. There has been significant research on the use of stem cells with and without biomaterial scaffolds to treat bone fractures due to their promising regenerative capabilities. However, the relative roles of exogenous vs. endogenous stem cells and their overall contribution to in vivo fracture repair is not well understood. The purpose of this study was to determine the interaction between exogenous and endogenous stem cells during bone healing. This study was conducted using a standardized burr-hole bone injury model in a mesenchymal progenitor cell (MPC) lineage-tracing mouse under normal homeostatic and osteoporotic conditions. Burr-hole injuries were treated with a collagen-I biomaterial loaded with and without labelled induced pluripotent stem cells (iPSCs). Using lineage-tracing, the roles of exogenous and endogenous stem cells during bone healing were examined. It was observed that treatment with iPSCs resulted in muted healing compared to untreated controls in intact mice post-injury. When the cell populations were examined histologically, iPSC-treated burr-hole defects presented with a dramatic reduction in endogenous MPCs and cell proliferation throughout the injury site. However, when the ovaries were removed and an osteoporotic-like phenotype induced in the mice, iPSCs treatment resulted in increased bone formation relative to untreated controls. In the absence of iPSCs, endogenous MPCs demonstrated robust proliferative and osteogenic capacity to undertake repair and this behaviour was disrupted in the presence of iPSCs which instead took on an osteoblast fate but with little proliferation. This study clearly demonstrates that exogenously delivered cell populations can impact the normal function of endogenous stem/progenitor populations during the normal healing cascade. These interactions need to be better understood to inform cell and biomaterial therapies to treat fractures.

## Introduction

Throughout the lifespan, bone continuously remodels in response to loading, metabolic changes, and damage (such as fractures). In humans, fractures to long bones typically heal within 3 to 4 months^1^. Skeletal stem/progenitor cells, such as mesenchymal stem/progenitor cells (MPCs) play an important role in bone healing as they have the ability to differentiate into many of the cell types involved in the healing cascade (e.g. chondrocytes, osteoblasts, and osteocytes) as well as influence the inflammatory niche (immunomodulation) during the repair process^2^.

Despite bone’s intrinsic capacity to regenerate post-injury, there are several instances in which bone is unable to heal on its own and these injuries can persist due to a variety of factors such as unstable fracture mechanics or diseases such as osteoporosis^3^. In these cases, a non-repairing defect called a fracture non-union may arise. Fracture non-unions are generally characterized by a lack of healing in 6–8 months, with no progressive signs of healing^4^ and it estimated that 100,000 fractures proceed to non-union in the USA alone^5^. Therefore, promoting the healing of fracture non-unions is a promising target for bone tissue engineering because of the defect’s inability to regenerate, as well as its relatively high clinical incidence.

Due to their promising regenerative capabilities, there has been significant research on the use of exogenous stem cells (adult and pluripotent) with and without biomaterial scaffolds to treat bone fractures^6–9^. However, despite the potential for stem cell therapy in fracture repair, there still remains a lack of research investigating the interaction of exogenous vs. endogenous stem/progenitor cells during the fracture healing process. As well, it is not yet clear through what mechanisms endogenous MPCs contribute to fracture healing; there is evidence that they differentiate and contribute to new tissue formation^10^, as well they may contribute indirectly by releasing growth factors/cytokines to promote healing^6,10,11^. To gain a better understanding of the cellular mechanisms through which bone heals, it is necessary to determine how exogenous and endogenous stem/progenitor cell populations interact with each other during healing in vivo*.* Therefore, the purpose of this study was to examine the interaction between exogenous (induced pluripotent stem cells, iPSCs) vs. endogenous (MPCs) cells during fracture repair to gain insight into these mechanisms with the goal of improving healing in fracture non-unions.

To assess healing, a highly reproducible burr hole injury was generated in the proximal tibia^7,12–14^, in conjunction with a lineage tracking murine model (*Hic1*^cre-ERT2^:*Rosa*^tdTomato^)^15–17^ wherein endogenous MPCs can be identified post-injury. In this mouse model the endogenous MPCs are permanently labelled with tdTomato post-tamoxifen induction^15,16^. To assess exogenous stem cells in the repair process, green fluorescent protein (GFP) tagged iPSCs embedded in a collagen-I biomaterial^14,18^ were transplanted into the burr hole defect site. The endogenous MPCs and exogenous iPSCs could then be tracked simultaneously during fracture healing with the dual fluorescent markers. In addition to tracking these cells post-fracture, we also employed an osteopenic/porotic model (ovariectomy/OVX) to gain a better understanding of the role of exogenous and endogenous stem cell populations in bone healing under intact homeostatic vs. non-homeostatic (disease) conditions.

## Materials and methods

### Ethics statement

All animal studies were performed in accordance with the recommendations in the Canadian Council on Animal Care Guidelines. The reporting of this data in the manuscript follows the recommendations in the ARRIVE guidelines. The University of Calgary Health Sciences Animal Care Committee approved all animal protocols and surgical procedures used in this study (Ethics ID# AC16-0043).

### iPSC generation

C57BL/6 embryos (day 12.5) were harvested, rinsed in Dulbecco’s phosphate buffered saline (DPBS), and macerated. After treatment with 0.25% trypsin, the resultant cell suspension was filtered to remove debris and plated in high glucose DMEM (Gibco), 10% FBS (Gibco), 1× MEM No-Essential Amino Acids (Gibco), 50 units/ml Penicillin–Streptomycin (Gibco). The resultant mouse embryonic fibroblasts (MEFs) were shipped Applied Biological Materials Inc. (Richmond, BC) for cellular reprogramming to produce iPSCs. MEFs were transduced with Lentiviruses SFFV-OCT4, -SOX2, and -KLF4. After one week, ESC-like colonies were transferred to fresh wells with feeders. Following two passages on feeder cells, mouse iPSCs were shipped to UCalgary. To incorporate GFP into the genome, CRISPR/Cas9 was used to insert a 6-kb transgene into the mouse safe harbor ROSA26 locus using homology-independent targeted integration (HITI) technique was used [136]. For each nucleofection reaction, CRISPR DNA 10 μl from 916 ng/μl and DNA 10 μl from 719 ng/μl Cas9 DNA construct mixed with 28.98 μl from 414 ng/μl GFP construct was used for iPSCs. All DNA constructs were dissolved in water. For each nucleofection reaction, constructs with three million iPSCs was used with nucleofection kit (mouse nucleofector kit, Lonza, Cat. No.VPH-1001) in program A-023 according to manufacturer’s instruction. Nucleofected iPSCs were grown on the gelatin and MEFs coated plates with ESC media for two days. Then FACS with SSEA1 marker was performed. iPSCs were digested enzymatically with 0.25% trypsin for 5 min in 37 °C. Single cells washed twice with cold DPBS and stained with conjugated SSEA1-PE with concentration of 1 μg/ml (Santa cruz: sc-21702 PE) for 15 min. Double positive for GFP and SSEA1 iPSCs were sorted into gelatin coated 96-well plates, one cell/well. Clones were sequenced to determine correct positioning and orientation of the transgene in the ROSA26 locus. To validate the cells remained as functional iPSCs, teratoma assays were undertaken in where 1.10^6^ cells were transplanted subcutaneously into the dorsal area of SCID-beige mice. Samples were fixed, processed and embedded in paraffin. They were sectioned to a thickness of 10 μM and stained with Safranin O.

### iPSC culture

Murine iPSCs were routinely cultured on mitotically inactivated mouse embryonic fibroblasts (MEFs) in T25 flasks (Fisher). Culture medium consisted of high glucose Dulbecco’s Modified Eagle Medium (DMEM, Lonza) supplemented with 1% non-essential amino acid, 1% Anti-Anti, 15% fetal bovine serum (FBS), and 0.1 mM β-mercaptoethanol (all Invitrogen). In order to maintain iPSC pluripotency, culture medium was supplemented with 1000 U/ml leukemia inhibitory factor (LIF). Cells were routinely passaged upon reaching approximately 80% confluence every third to fourth day and maintained in a humidified incubator with 5% CO_2_ at 37 °C. One passage before the cells were added to the collagen scaffold, they were cultured on gelatine (0.1%, Fisher) coated flasks to remove excess MEFs.

### Scaffold preparation

The collagen-I scaffold was prepared according to previously published methods^7,14,18^. Bovine fibrillar collagen I (3 mg/ml, PureCol, Advanced Biomatrix) was polymerized as a 3D gel. Briefly, 80% v/v 3 mg/ml type-I collagen solution was mixed with a 1 million cells/ml iPSC suspension and 20% v/v beta-glycerol phosphate (βGP, Sigma Aldrich) dissolved in 5 × concentrated Dulbecco’s modified Eagle’s medium (DMEM)^18^. Subsequently, 15% FBS (Invitrogen), 1% non-essential amino acids, 1% Anti-Anti, and 0.1 mM β-mercaptoethanol (all Invitrogen) was added^18^. The collagen-I/iPSC construct was distributed into a 96-well plate with a volume of 100 μl per well. The polymerized scaffold was placed in an incubator at 37 °C and 5% CO_2_ to pre-differentiate for 5 days prior to in vivo implantation in the animal model. For the collagen-only scaffold control, the above protocol was also carried out, however, the iPSCs were not added. A general overview of the process is shown in Supplementary Fig. S1.

### Real time polymerase chain reaction (RT-PCR)

The collagen-I scaffolds with iPSCs were collected at timepoints: 0, 1, 3, 5, 8, 11, 13, 15, 18 and 21 days post-seeding. Trizol (Invitrogen) was added and a 26 ½ gauge needled was used to lyse the collagen/cell matrix in the Trizol. RNA was isolated using the manufacture’s recommended protocol. cDNA was prepared using the High-Capacity cDNA Reverse Transcription Kit (ThermoFisher). RT-PCR was performed on an ABI QuantStudio 6 using probes again *Sp7* (Mm04209856_m1) and *Bglap* (Mm03413826_mH), *Ibsp* (Mm00492555_m1), *Sox9* (Mm00448840_m1), *Col2a1* (Mm01309565_m1), *Oct4* (Mm03053917_g1), *Nanog* (Mm02019550_s1) with 18S (Mm03928990_g1) as a housekeeping control.

### Viability assessment by flow cytometry

Cells were dissociated into single-cell suspensions with collagenase I treatment and subjected to flow cytometry using an Attune NXT and FlowJo software for analysis. At least ten thousand events were registered per sample, and analysis of whole cells was performed using appropriate scatter gates to avoid cellular debris and aggregates. Cells were stained using the Annexin-PI kit (ThermoFisher) following the manufactures instructions.

### Animal models

MPC lineage tracing mice (*Hic1*^cre-ERT2^:ROSA^tdTomato^) on a C57BL/6 background were provided by Dr. T. Michael Underhill (University of British Columbia). Both males and females ranging 8–12 weeks old, were used in this study. The experimental design for the bone injury in intact and ovariectomy (OVX) mice is depicted in Supplementary Fig. S2.

In this mouse model the endogenous MPCs were permanently labelled with tdTomato post-tamoxifen induction. Mice were anesthetized and received intraperitoneal injections of the active isomer of tamoxifen ((*Z*)-4-Hydroxytamoxifen, Sigma) dissolved in sterilized sunflower oil. Mice were injected with 100 μl tamoxifen solution (100 mg/kg) once a day over 4 days, followed by a 1 week waiting period to allow for recombination.

One week after the last tamoxifen injection, a burr-hole (non-critical size) injury was performed. The burr-hole injury was performed following procedures previously described by Taiani et al*.*^12,13,19^, which was modified from methods previously published by Uusitalo et al*.*^20^ Mice were anesthetized with veterinary isoflurane and 0.1 ml of buprenorphine was administered subcutaneously prior to surgery. A high-speed microdrill (Fine Science Tools) was used to drill a 0.7 mm diameter hole into the medullary cavity (without damaging the opposite side) of the metaphysis of the proximal tibia^19^.

In the homeostatic fracture model, mice were divided into three groups: untreated control, empty collagen I construct, and collagen I + iPSCs. Immediately following the burr-hole injury, the skin of untreated control mice was stapled to close the incision site. For the empty collagen I and collagen I + iPSC treated mice, 100 µl of gel with (100,000 iPSCs per mouse) or without cells, was implanted into the burr-hole defect. The incision site was then closed with staples and mice were returned to their cages and allowed to weight-bear immediately following surgery.

Similar to the experimental design of the homeostatic fracture healing model, Hic1^cre-ERT2^:ROSA^tdTomato^ received intraperitoneal tamoxifen injections once per day for 4 days. One week after the last tamoxifen injection, mice received OVX surgery. Briefly, mice were anesthetized and given 0.1 ml of buprenorphine prior to surgery. Both ovaries were located and excised. One week after OVX surgery, burr-hole surgery was performed. The OVX mice were divided into three groups: OVX untreated control, OVX empty collagen I construct, and OVX and collagen I + iPSCs.

### Histology and immunofluorescence

Tibiae were decalcified, processed and embedded in paraffin wax and cross-sectioned at 10 μm. Safranin-O/fast-green staining and immunofluorescence were performed. The specific markers used were: TdTomato, GFP, Anti-Mo/Rt Ki-67 eFluor 660 Clone SolA15 (Invitrogen, Ki67), Bone sialoprotein (BSP) Clone WVID1(9C5) (Developmental Studies Hybridoma Bank). Slides were treated with EverBrite™ Hardset Mounting Media with DAPI (Biotium), and imaged using the Zeiss Axioscan microscope.

### Tissue cytometry

For quantitative analysis, the area of interest was acquired as digital greyscale images. Cells of a given phenotype were identified and quantitated using the TissueQuest software (TissueGnostics), with cut-off values determined relative to the negative controls (non-stained and secondary alone controls). Gating and quantification of single/double positive cells were undertaken using these thresholds.

### X-ray microscopy (Xradia)

X-ray microscopy (XRM) imaging was employed on C57BL/6 mice (normal and OVX) to assess bone structure and callus formation within the injury area. The tibiae (including soft tissue and muscle) were harvested and fixed in 10% neutral buffered formalin (NBF) for 24 h. After 24 h, all soft tissue and muscle were removed from the bone. Samples were placed in 70% alcohol until XRM imaging. To prepare samples for XRM, the tibia was removed from the alcohol and secured using foam in an upright holder. PBS was used to ensure hydration during imaging. Three calcium hydroxyapatite (CHA) bone calibration phantoms (densities 50, 1000, and 1200 mg/cm^3^) were placed on top of a thin foam layer to sit flush and secured to the holder using tape. Low energy (40 kVp voltage, 3 W power) XRM scans were performed using a 4 × objective. The exposure time of each projection was 3 s, and 2001 projections were collected per rotation. Images were reconstructed to an isotropic voxel size of 4.9 μm. The raw data obtained from the XRM scan was processed using Amira software and custom SimpleITK scripts (v0.10.0, http://www.simpleitk.org/)^21^. All slices containing the burr hole defect were segmented, including the area up to where the cortical bone ends and all newly formed bone callus extending from the defect site (Supplementary Fig. S3). Regions of interest (ROIs) were placed in the CHA calibration phantoms avoiding edge artifacts using ITK-SNAP (v3.8.0, http://www.itksnap.org/)^22^. Average linear attenuation was computed in each ROI and a linear calibration equation was fit to calibrate the XRM images. For all calibrations, R^2^ > 99%. A density threshold of 800 mg/cm^3^ was used to segment fully mineralized tissue. Callus bone volume fracture (BV/TV) was computed as the number of fully mineralized voxels in the callus segmentation divided by the total number of voxels in the callus segmentation. Callus bone mineral density (BMD) was computed as the average density inside the callus segmentation. Callus renderings were generated using ParaView (5.7.0) software. Three filters were applied: (1) Threshold—at a minimum of 0.5, (2) Extract surface, and (3) Smooth—at 400 iterations.

### Statistical analysis

All data was analyzed using GraphPad Prism 9. All data sets containing more than two experimental groups were analyzed using a one-way analysis of variance (ANOVA) with a 95% confidence interval (α = 0.05) with a Fisher’s LSD post-hoc test. Data sets containing only two groups were analyzed using a two-tailed unpaired parametric t-test with a 95% confidence interval (α = 0.05).

## Results

### Validation of osteogenic differentiation (in vitro)

To validate that the modified iPSCs retained the ability to differentiate into all three germ layers, teratoma analysis was conducted and tissues derived from all three germ layers including bone was observed (Supplementary Fig. S4).

Collagen I scaffolds with iPSCs were collected over 21 days of differentiation in vitro*,* and assayed for expression of the osteogenic markers *Sp7, Bglap* and *Ibsp*; chondrogenic markers *Sox9* and *Col2a1*; pluripotent markers *Oct4* and *Nanog* (Supplementary Fig. S4). iPSCs seeded in collagen I scaffold (at 100,000 cells/100 μl) showed increased expression of *Sp7 Bglap* and *Ibsp. Sp7* expression peaked at day 1 of differentiation compared to *Bglap* and *Ibsp* which peaked at days 15 and 11 respectively (Supplementary Fig. S4). Chondrogenic markers were not detected for the first 7 days of differentiation and then both *Sox9* and *Col2A1* expression was observed, peaking at day 18 and 15 respectively (Supplementary Fig. S4). While pluripotent marker expression (Oct4 and Nanog) was detected in undifferentiated iPSCs, neither marker was detected by day 7 of differentiation (Supplementary Fig. S4). The viability of the cells in the collagen scaffold was also examined during the differentiation period using flow cytometry. The undifferentiated iPSCs were ~ 97% viable at the time they were seeding into the scaffolds and this decreased to ~ 72% after 5 days in the scaffold (timepoint of transplantation). By the end of the in vitro differentiation period ~ 15% of the cells were viable (Supplementary Fig. S4).

### Localization of MPCs in uninjured bone

Before examining the interaction of endogenous MPCs with exogenous iPSCs post-injury, the localization of lineage traced *Hic1*^+^ MPCs was examined in uninjured bone in mice 7 days post-tamoxifen induction (Supplementary Fig. S5). In uninjured bone, MPCs were localized to bone surfaces (periosteal and endosteal).Within the cortical bone, there were few *Hic1*^+^ MPCs and only a small fracture of these expressed BSP. However, within the bone marrow cavity, there was an increase in the *Hic1*^+^ population with approx. 10% of these cells expressing bone sialoprotein (BSP) and approx. 20% expressing Ki67 (Supplementary Fig. S5A–F).

### Localization of MPCs and iPSCs post-injury

**Figure 1:**
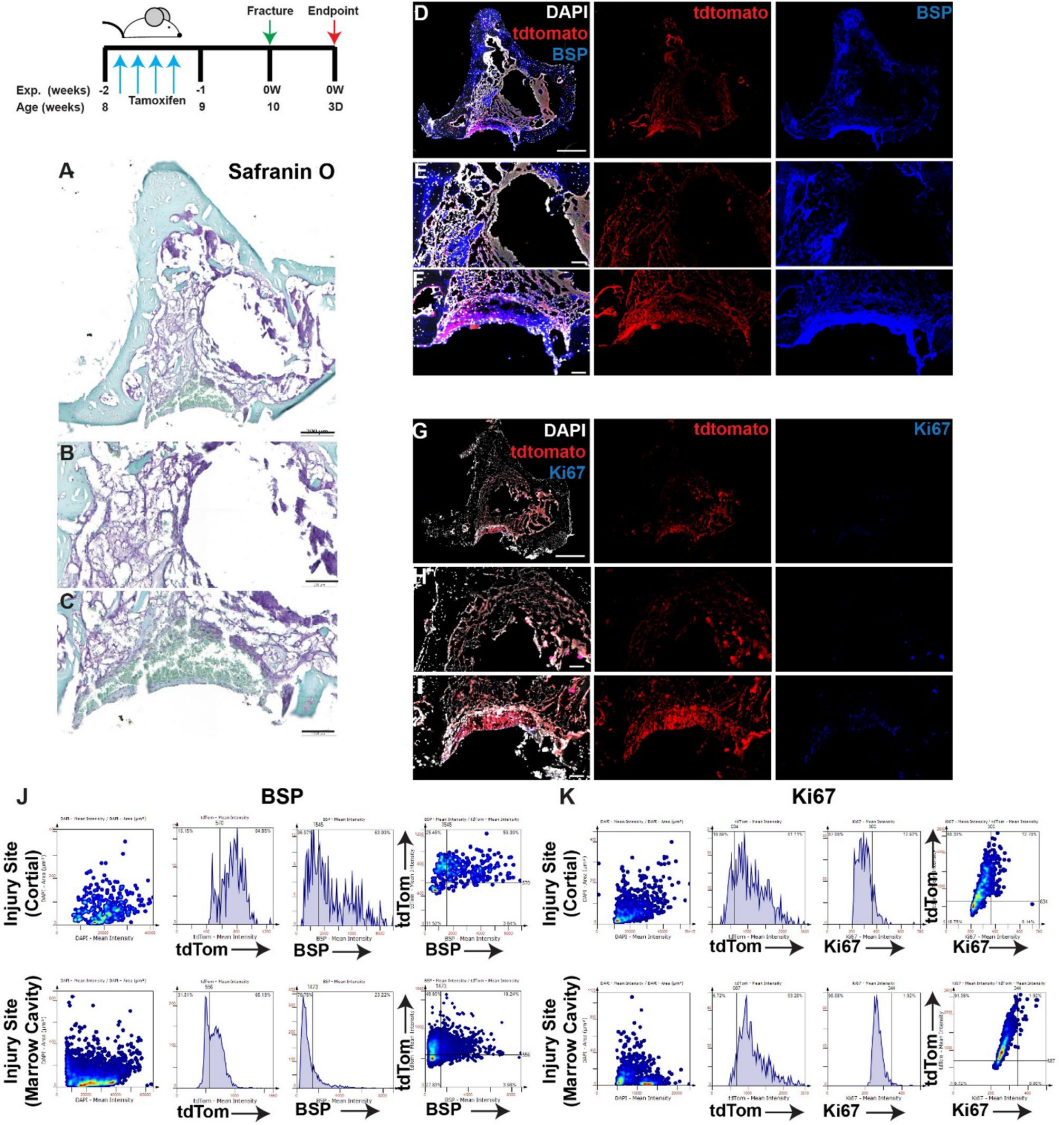
Lineage tracing of MPCs at 3 days post-injury without intervention. The localization of *Hic1*^+^ lineage traced MPCs (tdTomato **D**–**I**), BSP (**D**–**F**) and Ki67 (**G**–**I**) are presented. A representative Safranin O image shows the presence of proteoglycan staining within the injury site (**A**–**C**). Scale bars in (**A**,**D**,**G**) = 200 µm, scale bars in remaining figures = 100 µm. Representative tissue cytometry gates from the same groups (**J**,**K**).

**Figure 2:**
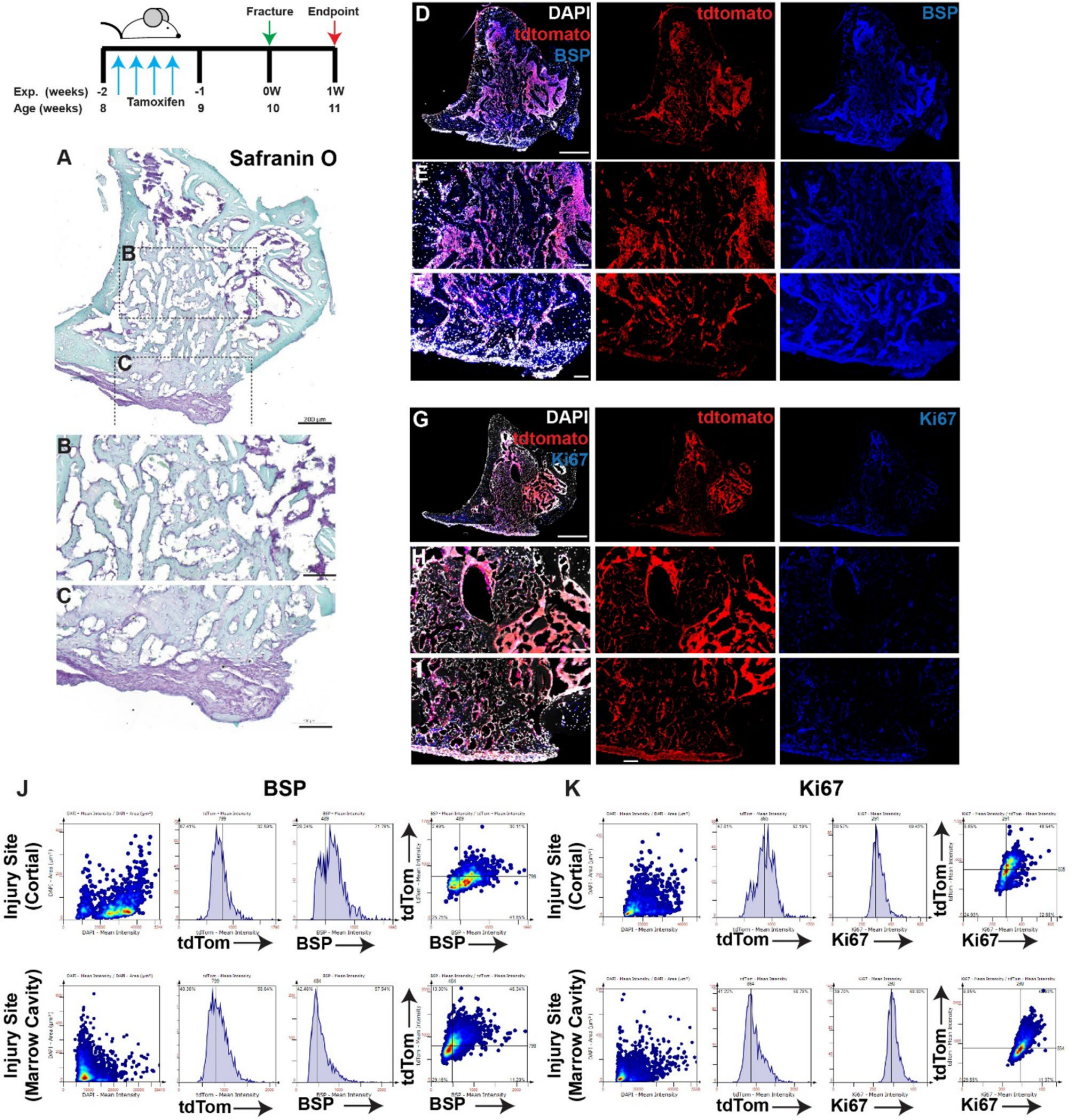
Lineage tracing of MPCs at 7 days post-injury without intervention. The localization of *Hic1*^+^ lineage traced MPCs (tdTomato **D**–**I**), BSP (**D**–**F**) and Ki67 (**G**–**I**) are presented. A representative Safranin O image shows the presence of proteoglycan staining within the injury site (**A**–**C**). Scale bars in (**A**,**D**,**G**) = 200 µm, scale bars in remaining figures = 100 µm. Representative tissue cytometry gates from the same groups (**J**,**K**).

At 3 (Fig. 1) and 7 (Fig. 2) days post-injury without treatment; lineage traced MPCs were enriched at the cortical region of the injury at both timepoint, and in the bone marrow cavity at day 7 (Figs. 1, 2). Co-localization of tdTomato and BPS within the marrow cavity, adjacent endosteal bone surfaces and the injury site with the cortical bone was observed at both timepoints (Figs. 1, 2D–F,J). While minimal cell proliferation (Ki67) was observed within the injury site at day 3, robust Ki67 staining was observed throughout the injury site, including the bone marrow cavity by day 7 (Figs. 1, 2G–I). At 3 days post-injury, only a minor proportion of tdTomato positive cells also expressed Ki67 (Fig. 1G–I,K), with a dramatic increase in double positive tdTomato and Ki67 cells observed by day 7 post-injury (Fig. 2G–I,K).

**Figure 3:**
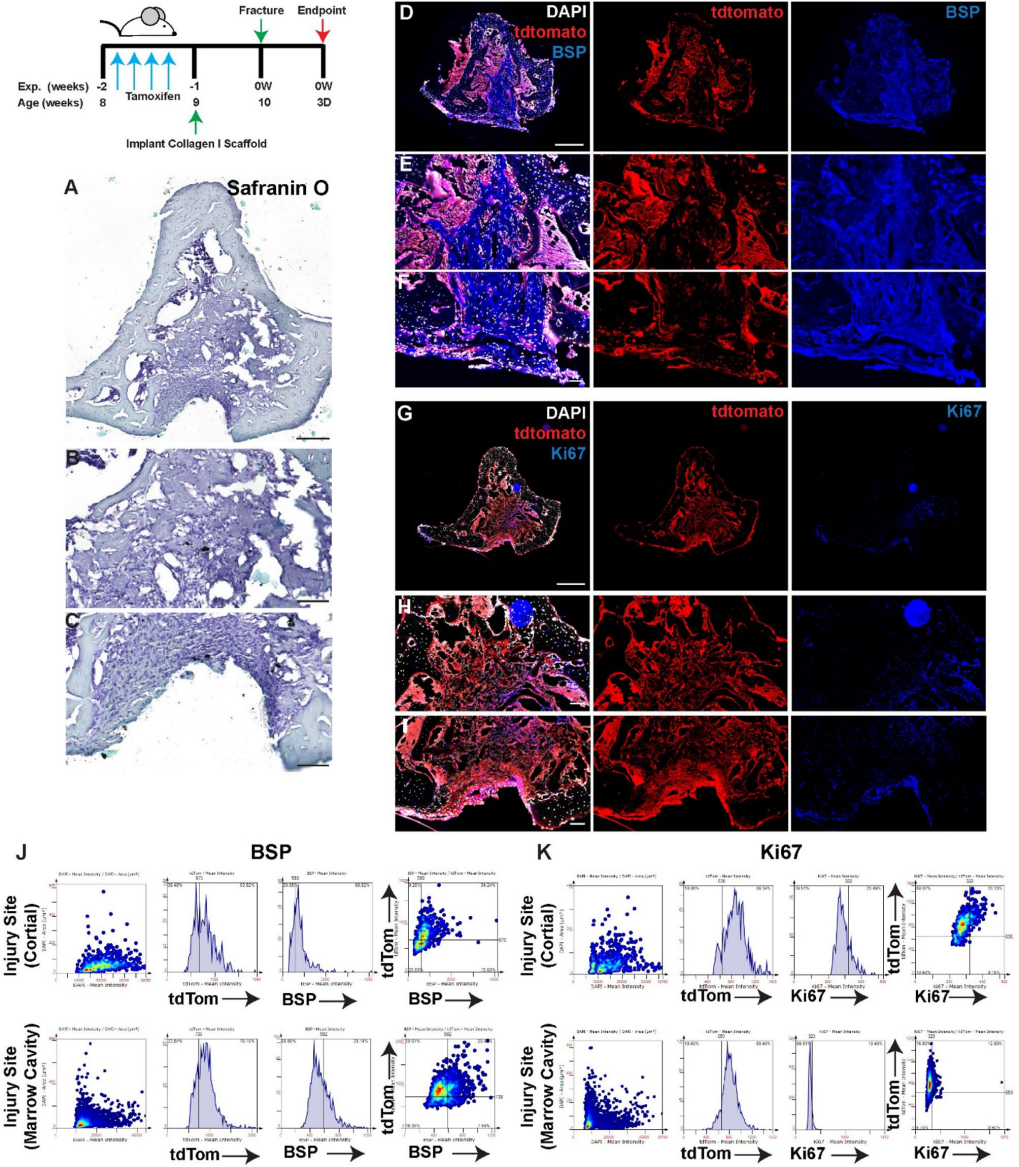
Lineage tracing of MPCs at 3 days post-injury with implantation of collagen scaffolds. The localization of *Hic1*^+^ lineage traced MPCs (tdTomato **D**–**I**), BSP (**D**–**F**) and Ki67 (**G**–**I**) are presented. A representative Safranin O image shows the presence of proteoglycan staining within the injury site (**A**–**C**). Scale bars in (**A**,**D**,**G**) = 200 µm, scale bars in remaining figures = 100 µm. Representative tissue cytometry gates from the same groups (**J**,**K**).

**Figure 4:**
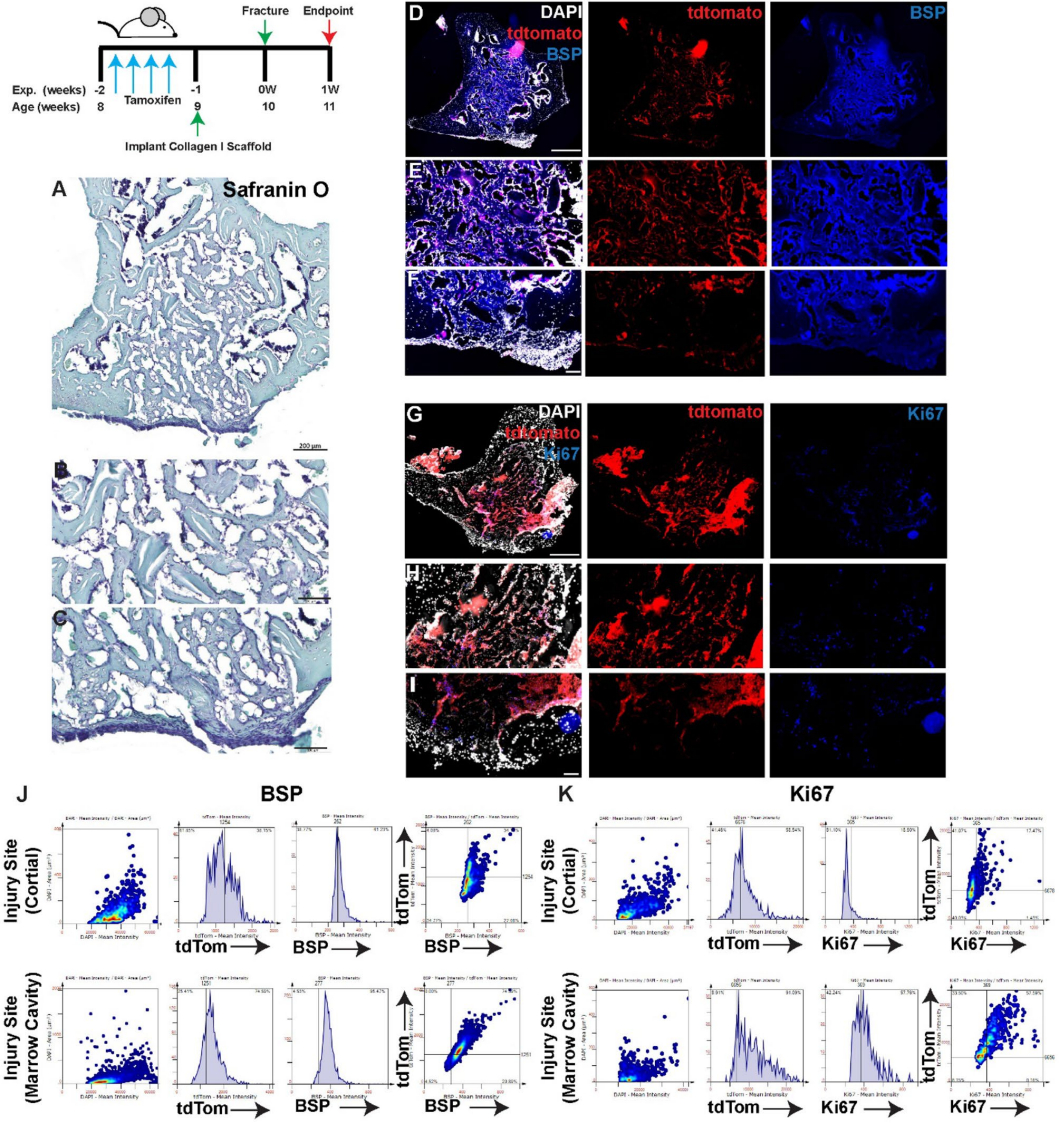
Lineage tracing of MPCs at 7 days post-injury with implantation of collagen scaffolds. The localization of *Hic1*^+^ lineage traced MPCs (tdTomato **D**–**I**), BSP (**D**–**F**) and Ki67 (**G**–**I**) are presented. A representative Safranin O image shows the presence of proteoglycan staining within the injury site (**A**–**C**). Scale bars in (**A**,**D**,**G**) = 200 µm, scale bars in remaining figures = 100 µm. Representative tissue cytometry gates from the same groups (**J**,**K**).

When the injury was treated with collagen I scaffolds alone (no iPSCs) (Figs. 3, 4A–C), lineage traced MPCs were observed within the cortical injury site, marrow cavity and endosteal surfaces as well as the periosteal surface of the bone at both day 3 and day 7 post injury (Figs. 3, 4D–F). Robust BSP staining was observed throughout the cortical injury site and within the bone marrow cavity at both timepoints (Figs. 3, 4D–F). While few Ki67^+^ cells were observed within the cortical injury site at either timepoint, Ki67^+^ cells were observed in the marrow cavity at 3 days post-injury with an apparent reduction in positive cells by day 7 post-injury (Figs. 3, 4G–I). At both 3 and 7 days post-injury ~ 40–50% of *Hic1*^+^ cells also expressed BSP and ~ 20% of this *Hic1* lineage traced population was proliferative within the cortical injury site (Figs. 3, 4J,K). Within the bone marrow cavity ~ 25% of *Hic1*^+^ cells also expressed BSP and ~ 10% of this *Hic1* lineage traced population was proliferative at 3 days post-injury within the cortical injury site (Fig. 3J,K). By 7 days post-injury (Fig. 4A–C), nearly all *Hic1*^+^ cells also expressed BSP and ~ 60% of this cell population also expressed Ki67 (Fig. 4J,K).

**Figure 5:**
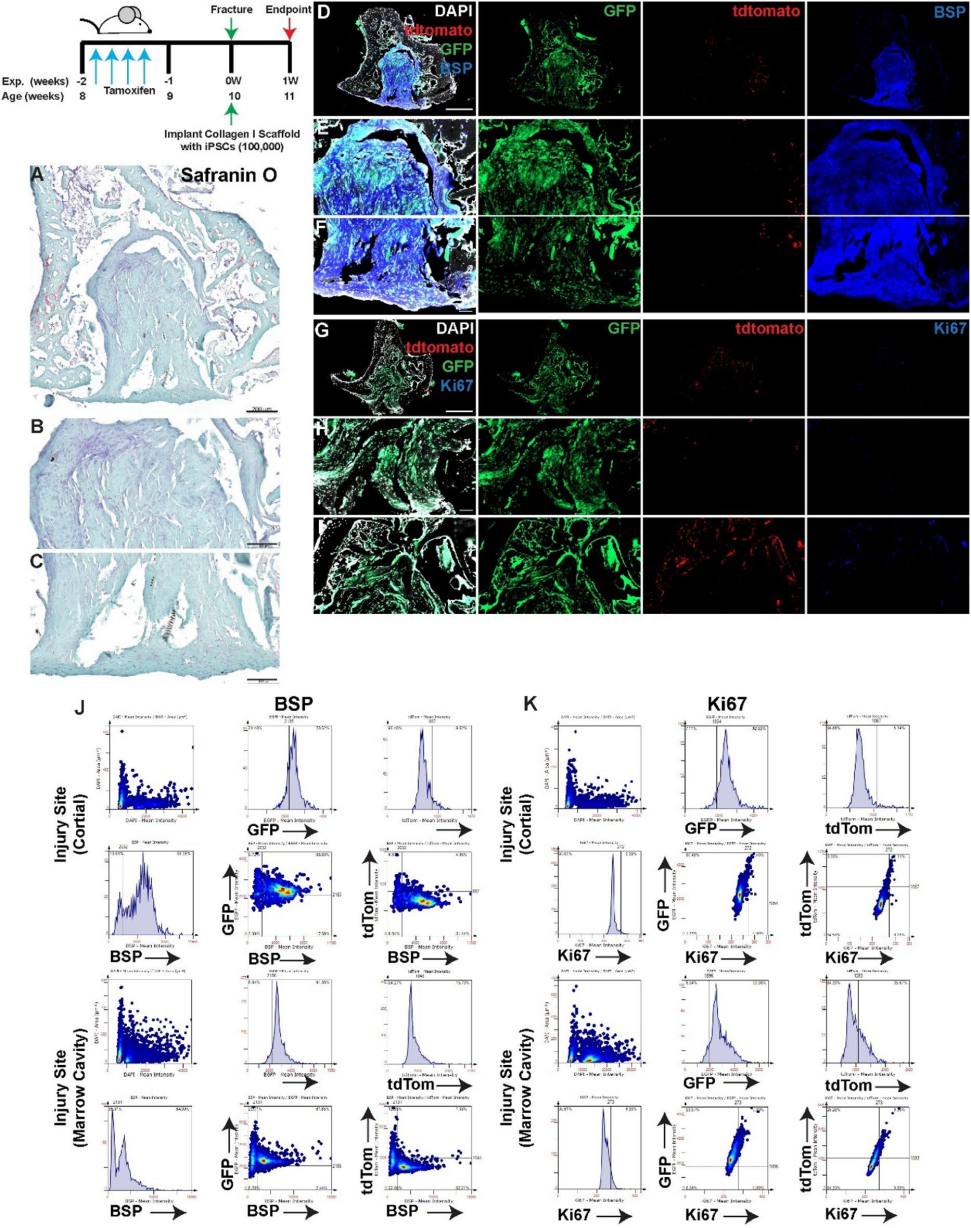
Lineage tracing of MPCs in injuries treated with iPSCs within collagen I scaffolds at 3 days post-injury. Collagen I scaffolds containing 100,000 iPSCs were injected into injuries and animals were sacrificed at 3 days post-treatment (**A**,**B**). The localization of iPSCs (GFP) and *Hic1*^+^ lineage traced MPCs (tdTomato) were examined in relation to BSP (**D**–**F**) or Ki67 (**G**–**I**) staining.. Scale bars in (**A**,**D**,**G**) = 200 µm, scale bars in remaining figures = 100 µm. Representative tissue cytometry gates from the same groups (**J**,**K**).

**Figure 6:**
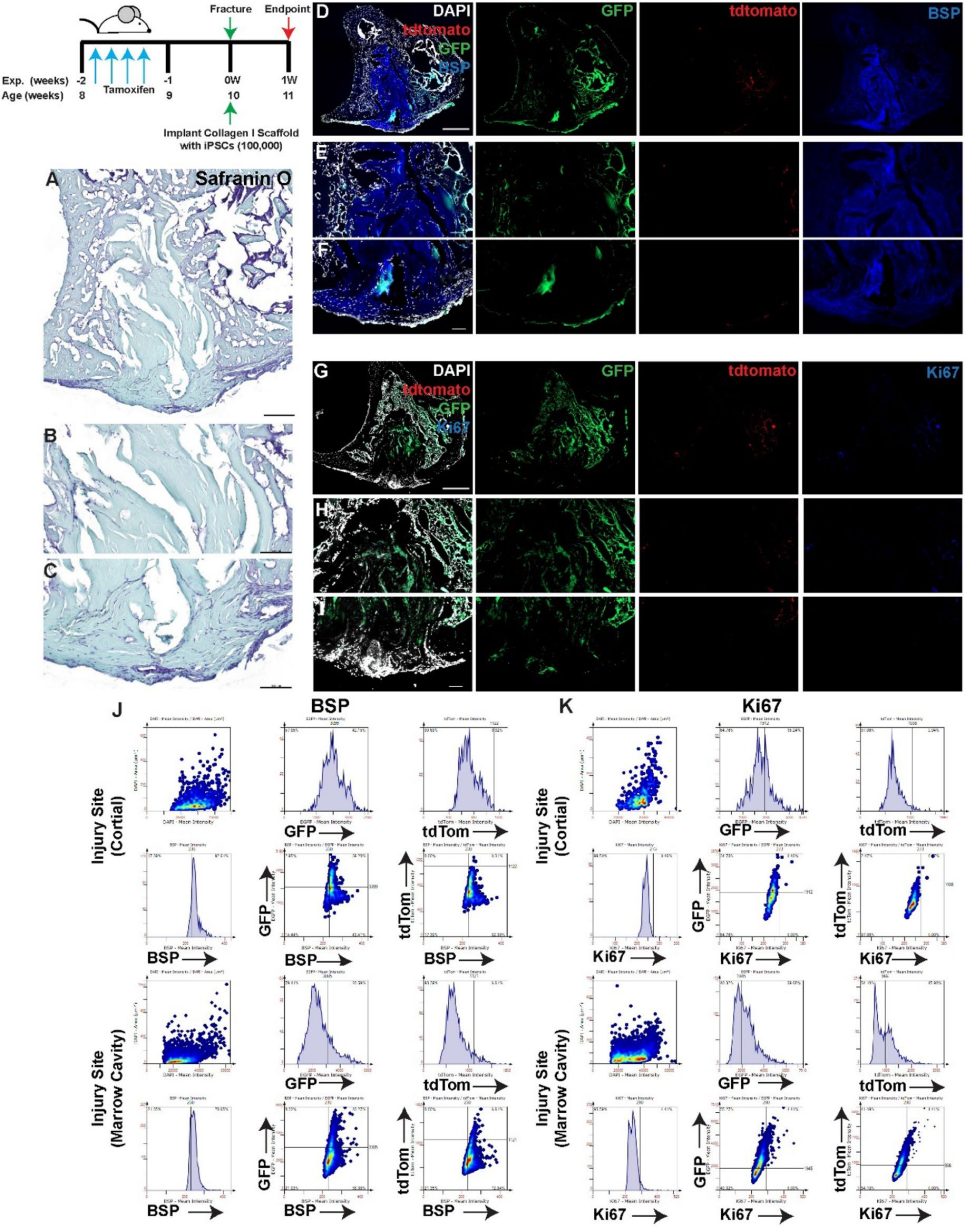
Lineage tracing of MPCs in injuries treated with iPSCs within collagen I scaffolds at 7 days post-injury. Collagen I scaffolds containing 100,000 iPSCs were injected into injuries and animals were sacrificed at 7 days post-treatment (**A**,**B**). The localization of iPSCs (GFP) and *Hic1*^+^ lineage traced MPCs (tdTomato) were examined in relation to BSP (**D**–**F**) or Ki67 (**G**–**I**) staining.. Scale bars in (**A**,**D**,**G**) = 200 µm, scale bars in remaining figures = 100 µm. Representative tissue cytometry gates from the same groups (**J**,**K**).

When collagen gels were loaded with iPSCs and implanted into the injury site, robust GFP expression was observed at the site of cortical injury as well as within the marrow cavity at 3 days post-injury (Fig. 5A–I) BSP staining was observed throughout the entire injury area and co-localized with both GFP (iPSC) and tdTomato (lineage traced MPCs) positive cells (Fig. 5D–F,J). In contrast to untreated and collagen I alone treated injuries, there was minimal Ki67 staining observed within the cortical or marrow cavity regions with little to no co-expression within GFP or tdTomato positive cells (Fig. 5G–I,K). By 7 days post-injury (Fig. 6A–I), GFP staining was still observed throughout the injury site, yet enriched in the bone marrow cavity vs. the cortical injury site. Few to no tdTomato cells were observed. BSP staining was also observed throughout the injury and co-localization with the GFP signal was detected (Fig. 6D–F,J). Minimal Ki67 expression was detected and only few GFP or tdTomato cells expressed Ki67 (Fig. 6G–I,K).

### Quantification of MPCs and iPSCs post-injury

The cell markers used in the study were quantified to determine if the numbers or fates of endogenous (MPCs) progenitor populations changed post-injury within the cortical injury area and/or the marrow cavity with or without the addition of exogenous iPSCs (Supplementary Fig. S6). In the untreated controls we observed an increase in the number of cells, MPCs (tdTomato), proliferating MPCs (tdTomato^+^Ki67^+^) and MPCs taking on an osteoblastic fate (tdTomato^+^BSP^+^) from day 3 to day 7 post-injury in the cortical and marrow cavity regions (Supplementary Fig. S6A,B). When collagen I scaffolds were introduced, there appeared to be a bias towards differentiation vs. proliferation as fewer tdTomato^+^Ki67^+^ cells were observed yet there was still an increase in tdTomato^+^BSP^+^ cells within the injury site (Supplementary Fig. S6C,D). With the addition of exogenous iPSCs in the collagen I scaffold, cell proliferation within the entire injury site was muted, while BSP positive cells were still observed. However, it appears that the majority of these BSP positive cells were derived from the exogenous iPSCs with little contribution to the BSP^+^ population from the endogenous MPCs (Supplementary Fig. S6E,F).

### Localization of MPCs in uninjured bone post-OVX

To gain a better understanding of how endogenous MPCs react to injury under non-homeostatic conditions, the burr-hole injury was induced in osteopenic/porotic mice (post-OVX). Immunofluorescence was used to identify the location of lineage traced MPCs at 7 days post-injury. The lineage traced MPCs were observed within the cortical bone and bone marrow cavity with staining also observed at bone surfaces (periosteal and endosteal). tdTomato co-localization with BSP and Ki67 was observed in marrow cavity and tdTomato co-localization with BSP was also observed within the cortical bone. However, minimal Ki67 expression or co-localization with tdTomato was observed in cortical bone (Supplementary Fig. S7A–H).

### Localization of MPCs post-injury in OVX mice

**Figure 7:**
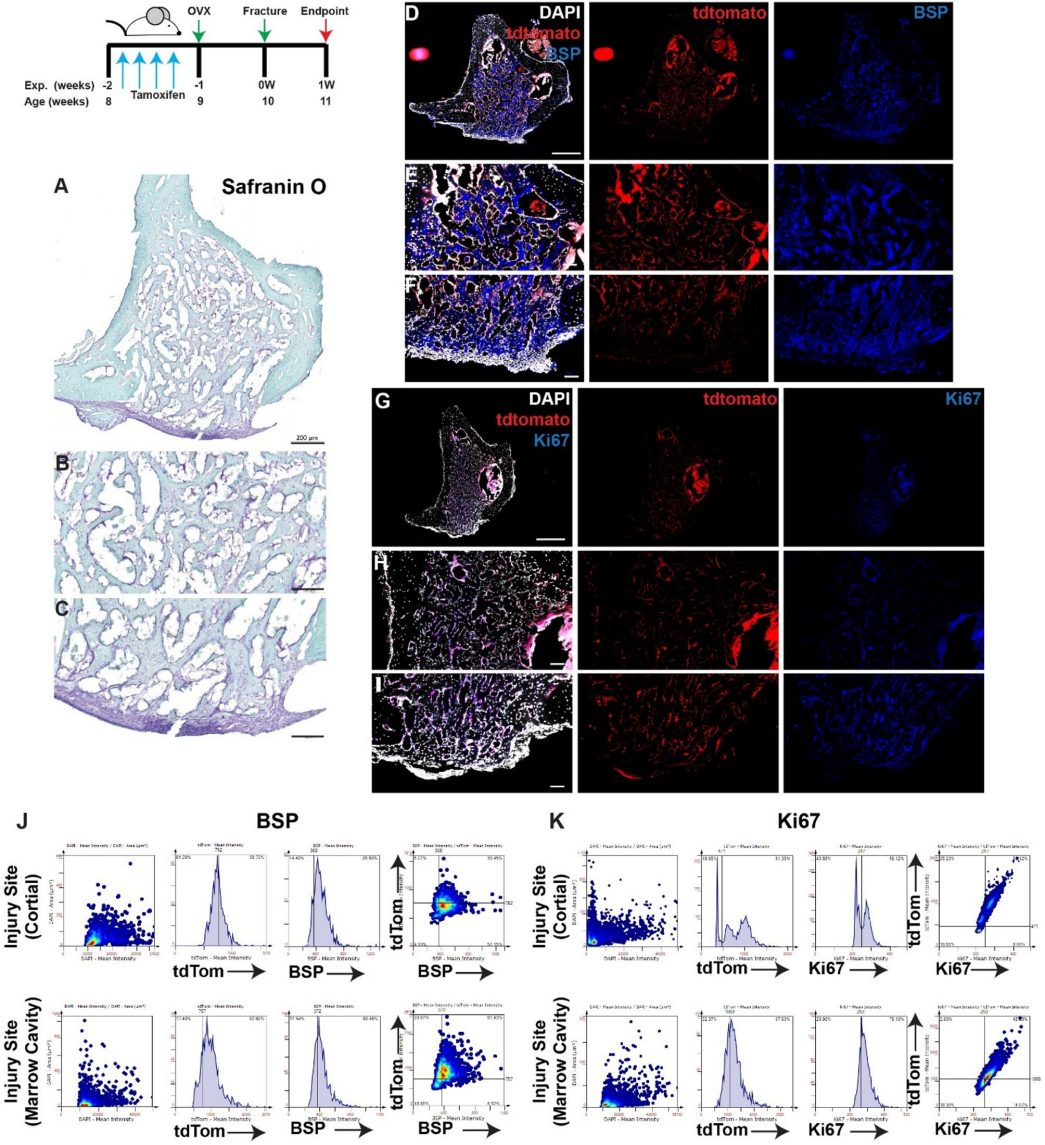
Lineage tracing of MPCs in OVX mice at 7 days post-injury without intervention. The localization of *Hic1*^+^ lineage traced MPCs (tdTomato **D**–**I**), BSP (**D**–**F**) and Ki67 (**G**–**I**) are presented. A representative Safranin O image shows the presence of proteoglycan staining within the injury site (**A**–**C**). Scale bars in (**A**,**D**,**G**) = 200 µm, scale bars in remaining figures = 100 µm. Representative tissue cytometry gates from the same groups (**J**,**K**).

At 7 days post-injury and 14 days post-OVX (in the absence of collagen I scaffolds ± iPSCs), lineage traced MPCs were observed within the cortical bone injury site in addition to the bone marrow cavity (Fig. 7A–I). Co-localization of tdTomato with BSP or Ki67 was observed throughout the injury site (Fig. 7A–K).

**Figure 8:**
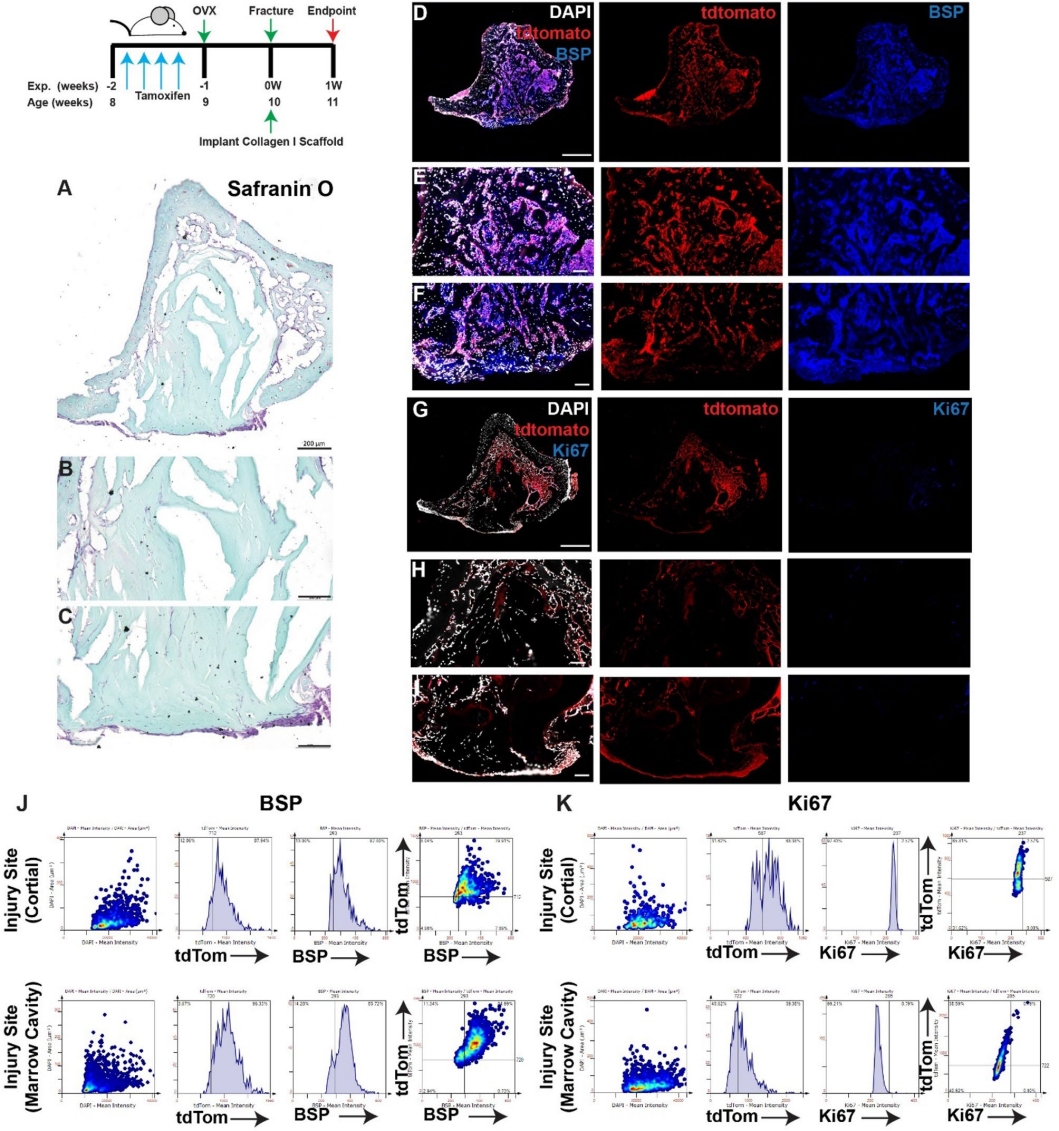
Lineage tracing of MPCs in OVX mice at 7 days post-injury with implantation of collagen scaffolds. The localization of *Hic1*^+^ lineage traced MPCs (tdTomato **D**–**I**), BSP (**D**–**F**) and Ki67 (**G**–**I**) are presented. A representative Safranin O image shows the presence of proteoglycan staining within the injury site (**A**–**C**). Scale bars in (**A**,**D**,**G**) = 200 µm, scale bars in remaining figures = 100 µm. Representative tissue cytometry gates from the same groups (**J**,**K**).

When OVX mice were treated with collagen I scaffolds alone (no iPSCs) (Fig. 8)) robust tdTomato expression was observed throughout the injury site (Fig. 8A–I). BSP was also observed throughout the entire injury site with intense staining observed in both the cortical and marrow cavity regions (Fig. 8A–F). While Ki67^+^ staining was also observed throughout the injury site, staining was enriched in the cortical injury area (Fig. 8G–I). Co-localization of tdTomato with BSP and Ki67 was observed in the cortical and marrow cavity regions (Fig. 8J–K).

**Figure 9:**
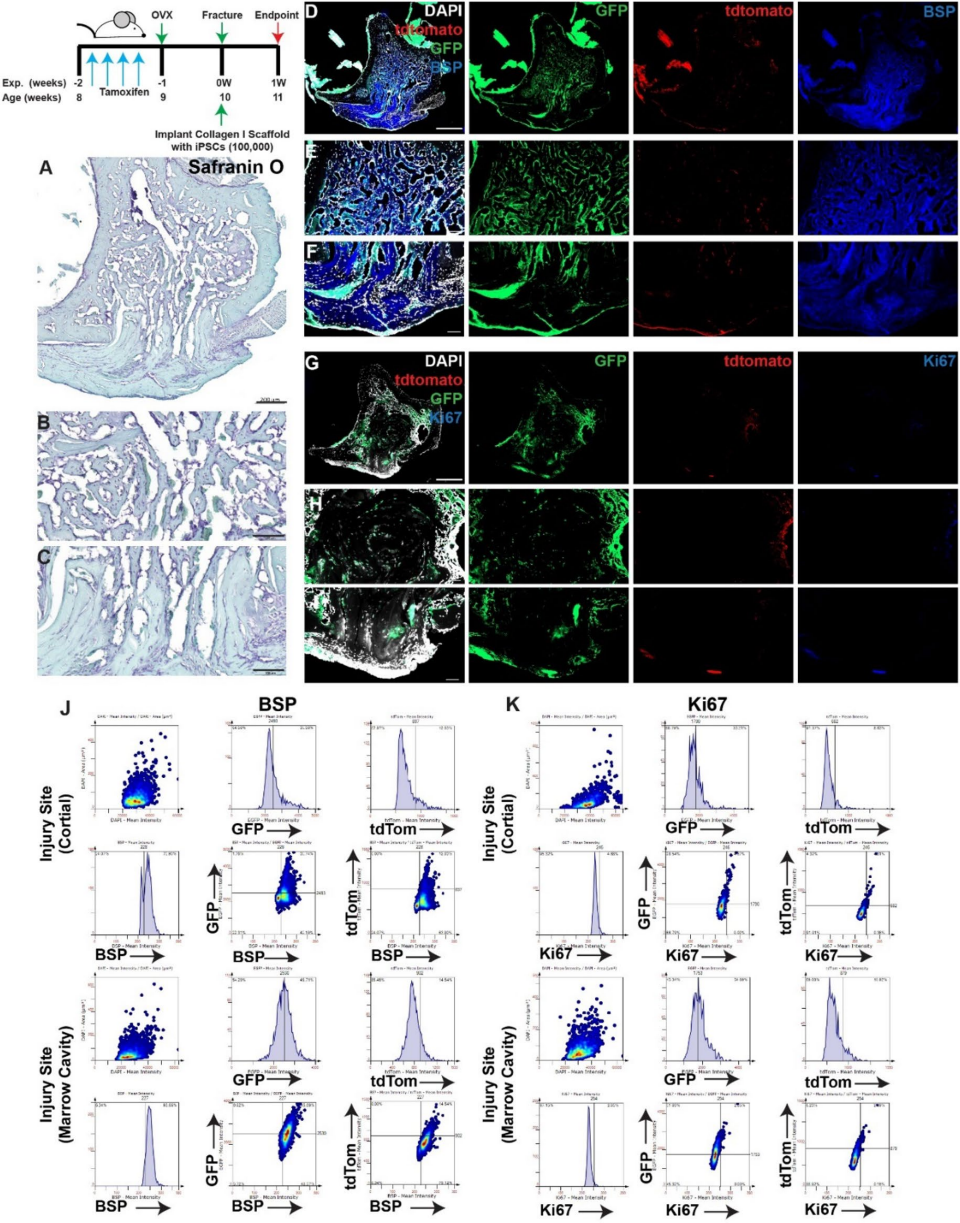
Lineage tracing of MPCs in OVX mice treated with iPSCs within collagen I scaffolds at 7 days post-injury. Collagen I scaffolds containing 100,000 iPSCs were injected into injuries at animals were sacrificed at 7 days post-treatment (**A**,**B**). The localization of iPSCs (GFP) and *Hic1*^+^ lineage traced MPCs (tdTomato) were examined in relation to BSP (**D**–**F**) or Ki67 (**G**–**I**) staining.. Scale bars in (**A**,**D**,**G**) = 200 µm, scale bars in remaining figures = 100 µm. Representative tissue cytometry gates from the same groups (**J**,**K**).

When collagen scaffolds were loaded with iPSCs and implanted into the injury site, GFP expression was observed in both the cortical injury area and within the marrow cavity (Fig. 9A–I). Similar to when iPSCs were injected into the injuries of intact mice, little to no tdTomato staining was observed. Yet, BSP staining was still observed throughout the injury site and co-localized with GFP (iPSC) expression (Fig. 9J). Little to no Ki67 staining was observed in the injury site (cortical or marrow cavity regions) and minimal co-localization of Ki67 was observed with GFP expression (Fig. 9G–I,K).

### Quantification of MPCs and iPSCs post-fracture in OVX mice

The cell markers used in the study were quantified in OVX mice (Supplementary Fig. S8). In the untreated injuries, there was an increase in all cell types examined relative to the non-injured control within the cortical and marrow cavity regions (Supplementary Fig. S8A,B). With the addition of the collagen scaffold (alone) there was a nearly complete loss of proliferative cells (including proliferative MPCs), with a concurrent increase in MPCs that took on an osteoblastic fate (tdTomato^+^BSP^+^) (Supplementary Fig. S8C,D). When iPSCs were introduced into the injury site, proliferation was again inhibited and the BSP^+^ cells in the cortical and marrow cavity regions were primary derived from the exogenous iPSCs (Supplementary Fig. S8E,F).

### Quantification of MPCs and iPSCs post-injury in normal vs. OVX mice

**Figure 10:**
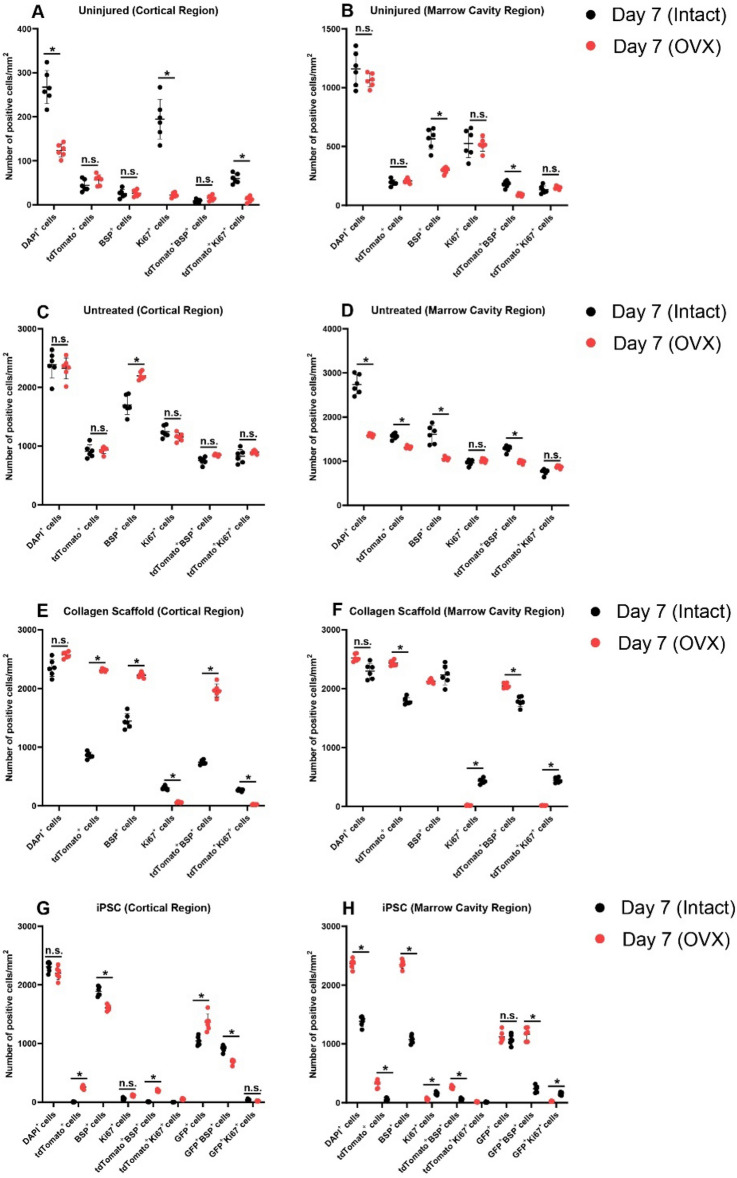
Quantification of cell populations within the injury site of intact vs OVX mice. Tissue cytometry data was quantified and the results examined statistically. The number of DAPI^+^, tdTomato^+^, BSP^+^, Ki67^+^, tdTomato^+^BSP^+^, tdTomato^+^Ki67, GFP^+^ iPSCs, GFP^+^Ki67^+^ GFP^+^BSP^+^ cell populations were quantified in uninjured (**A**,**B**) untreated (**C**,**D**), collagen scaffold (**E**,**F**) and collagen scaffold plus iPSC (**G**,**H**) treatment groups. n.s. = not significant. *p < 0.05.

To examine if the loss of bone homeostasis (OVX) impacted the behaviour the endogenous and/or exogenous stem/progenitor populations, the cell markers used in the study were quantified (Fig. 10). A number of significant differences were observed in the uninjured and injured treatment groups. In uninjured bone, there was a decreased in the total number of cells (DAPI^+^) in the cortical bone in addition to a decrease in proliferative cells in the bone post-OVX (Fig. 10A). In the marrow cavity of uninjured OVX bone, there was a decrease of the number of cells expressing BSP and lineage traced MPCs expressing BSP (tdTomato^+^BSP^+^) (Fig. 10B**)** In untreated injuries, the only difference within the cortical area was an increase in the number of BSP^+^ cells in the OVX mice (Fig. 10C). In the marrow cavity of these OVX mice, there was a decrease in MPCs, BSP^+^ cells and lineage traced MPCs expressing BSP (Fig. 10D). There were no differences in proliferation in the untreated mice.

When the collagen I scaffolds were implanted into the injury site, there was a dramatic increase in the amount of MPCs, BSP^+^ cells and MPCs expressing BSP in the cortical injury site of OVX mice (Fig. 10E). Interestingly, there was a decrease in the number proliferative cells in the cortical region and increase of proliferative cells in the marrow cavity of OVX mice (Fig. 10E,F). The most striking differences between intact and OVX mice that received iPSCs was in the marrow cavity in where there was a dramatic decrease in the total number of cells and BSP^+^ cells in mice that underwent OVX (Fig. 10H).

### Quantification of bone parameters within the injury site

Bone parameters from X-ray microscopy were quantified to determine the volume of new bone filling the defect site and the quality of bone formed. The bone volume fraction (BV/TV) and bone mineral density (BMD) were calculated and quantified in all mice (Supplementary Fig. S9). There was a significant decrease in BV/TV at 3 days post-injury followed by a significant increase by day 7. The same trend was observed in the presence of the collagen scaffold (Supplementary Fig. S9A). When iPSCs were introduced, a dramatic reduction in BV/TV was observed at days 3 and 7 post-injury (Supplementary Fig. S9A). The same trends were observed in BMD for thee mice, treatment groups and timepoints (Supplementary Fig. S9C). When intact vs. OVX mice were compared, a decreased in BV/TV and BMD in the uninjured OVX bone was observed as expected (Supplementary Fig. S9B,D). There were no differences observed in the untreated and collagen I scaffold alone groups in terms of BV/TV, but both groups showed decreased BMD in the OVX mice (Supplementary Fig. S9B,D). A significant increase in BV/TV was observed in OVX mice treated with iPSCs over intact mice, yet, the BMD values in the mice showed the opposite effect (Supplementary Fig. S9B,D).

## Discussion

This study was undertaken to gain a better understanding of the interactions between endogenous and exogenous stem/progenitor cells during bone healing. Using a highly reproducible model of bone injury (e.g. burr-hole) in intact/normal and osteoporotic/osteopenic (OVX) mice, we were able to demonstrate that there is a partial inhibition of endogenous MPC recruitment/function when exogenous iPSCs are delivered. To our knowledge, this is the first time this effect has been shown and this observation requires further investigation to determine if changing the mode of delivery and/or the dosage of exogenous cells can mitigate this inhibition and/or possible even result in synergy between endogenous and exogenous populations.

While it is important to recognize that our study design was not undertaken to optimize/improve fracture healing with iPSCs, we were able to demonstrate that delivery of iPSCs within an osteogenic-modified collagen I scaffold resulted in improved fracture healing in an impaired mouse model of bone homeostasis (OVX). These results align with previously published studies from our lab^14^ and others^23^ that show pluripotent stem cells (mouse or human) can facilitate fracture repair in impaired models. In the normal mice, the untreated burr-hole fracture filled with bone very quickly, as expected^7,12,24–26^. It was interesting however, to find that treatment with collagen I scaffold with or without iPSCs in an intact mouse impaired the normal healing process and resulted in equivalent or poorer outcome measures. One potential explanation of this in the physical presence of the dense scaffold that may have impeded cell recruitment and/or remodelling to the injury site. Aside from the physical interference of the collagen, it is quite possible that other osteo-chondral progenitor recruitment was impaired in the presence of iPSCs and/or other cell populations required for normal bone repair/homeostasis (such as osteoclasts). Furthermore, in these iPSC treated injuries, the bone formed within the injury site presented with an immature morphology lacking defined trabeculated structure compared to the other treatment groups. Yet, we cannot be sure that the iPSC themselves directly contributed to this immature bone phenotype, or simply delayed the natural healing process in these mice. Aside from a decrease in lineage traced MPCs within the defects that received iPSCs, we also observed minimal BSP and Ki67 staining in the MPC population compared to the other treatment groups (including collagen alone). This suggests that it was iPSCs that changed the behaviour of the endogenous progenitors, effectively inhibiting their proliferative and osteogenic differentiation capacity in this injury environment and this likely compromised their ability to effectively heal the burr-hole defect. In summary, these burr-hole defects treated with iPSCs formed less bone than untreated control and collagen treated groups, which appeared to be a function of fewer MPCs, committed osteo-progenitors/osteoblasts (BSP^+^) and proliferating cells in general (Ki67^+^). This result is quite interesting, since it has been previously demonstrated that transplanted iPSCs have the ability to increase the proliferation of endogenous cells to effect repair^27,28^. A study specifically designed to address this observation directly would need to be undertaken to determine why this is not occurring during the normal bone injury repair process.

Understanding the implications of using iPSCs within the bone healing process is essential to developing evidence-based therapies in the future. However, it is also critical to determine how these cells interact with the endogenous populations in an impaired model of bone fracture healing (OVX); as this environment better mimics the clinical situation wherein these types of therapies would be employed. The collagen scaffold I plus iPSC treated group demonstrated increased BV/TV vs. the untreated controls but showed no differences in total BMD. Therefore, it would appear that treatment with iPSCs was more effective in forming new bone in an impaired model of bone healing. This may suggest an osteoclast-iPSC interaction is occurring in the intact mice that is also inhibiting the natural repair response and that this interaction is interrupted in the OVX model. It is also possible that the transplanted iPSCs may be taking on an osteoclastic fate as it has been observed that iPSC osteoblastic/osteoclastic lineages are more plastic in iPSCs^23^. Another interesting observation was that in OVX mice treated with the collagen scaffold devoid of cells presented with very little bone regeneration (less BV/TV vs. iPSCs) and the bone that was present appeared to be less mature and lacked defined trabeculation. Furthermore, the entire injury site in these mice stained intensively positive for BSP. We confirmed that this was not a consequence of non-specific secondary antibody binding. This suggests that the BSP signal could be coming for other sources within the bone or systemically. It is well documented that BSP is present within the blood and these levels can be elevated due to diseases^29,30^ such as osteoporosis^31,32^. Therefore, it is possible that the collagen I scaffold on its own is acting as a ‘sink’ for increased systemic BSP levels due to the OVX, but it remains unclear why we would not have observed this in the iPSC treatment group, unless the systemic levels of BSP were decreased in this group. ELISA analysis of the serum would shed light on this hypothesis.

As with any scientific research study, limitations exist in the present study. While *Hic1* has been demonstrated to be a robust marker of MPCs^15–17^, by solely examining *Hic1* expressing cells, we likely did not account for all subpopulations of MPCs present in the adult mouse. For instance, it would be interesting to determine if the *Prx1*^33^ lineage displayed the same behaviour during facture repair with or without iPSCs. Another potential limitation/confounder is the day 7 timepoint which was selected based on previous findings that trabecular bone is formed in the medullary cavity one-week post-burr-hole surgery in normal and OVX mice^14^. To develop a more complete understanding of bone fracture healing in the injury site it would be valuable to evaluate later timepoints and/or using more complex fracture models^34^.

## Conclusion

In conclusion, the introduction of induced pluripotent stem cells (iPSCs) into a bone injury in intact mice led to an inhibition of the repair response, but increased the amount of bone formed in impaired (OVX) mice. Specifically, the introduction of iPSCs into the injury site in intact mice resulted in decreased bone formation, reduced endogenous MPC recruitment, proliferation and differentiation. Furthermore, the bone healing that was observed in OVX mice treated with iPSCs was not a result of increased recruitment or osteogenic differentiation of endogenous MPCs, but likely through the differentiation of these iPSCs into osteoblasts. Additional research is required to better elucidate the direct and indirect mechanisms through which iPSCs participate in bone healing so that they can be safely employed in evidence-based therapy for the treatment of difficult-to-heal non-union bone fractures.

## Supplementary Information

Supplementary Figures.

## Data Availability

The data that support the findings of this study are available from the corresponding author upon reasonable request.
