# Supplementary material for: Exogenously delivered iPSCs disrupt the natural repair response of endogenous MPCs after bone injury
Source: Sci Rep. 2023 Jun 9;13:9378. doi: 10.1038/s41598-023-36609-z (PMC10256810; doi:10.1038/s41598-023-36609-z)
Supplement: Supplementary file 1 — Supplementary Figures. [file 41598_2023_36609_MOESM1_ESM.docx]

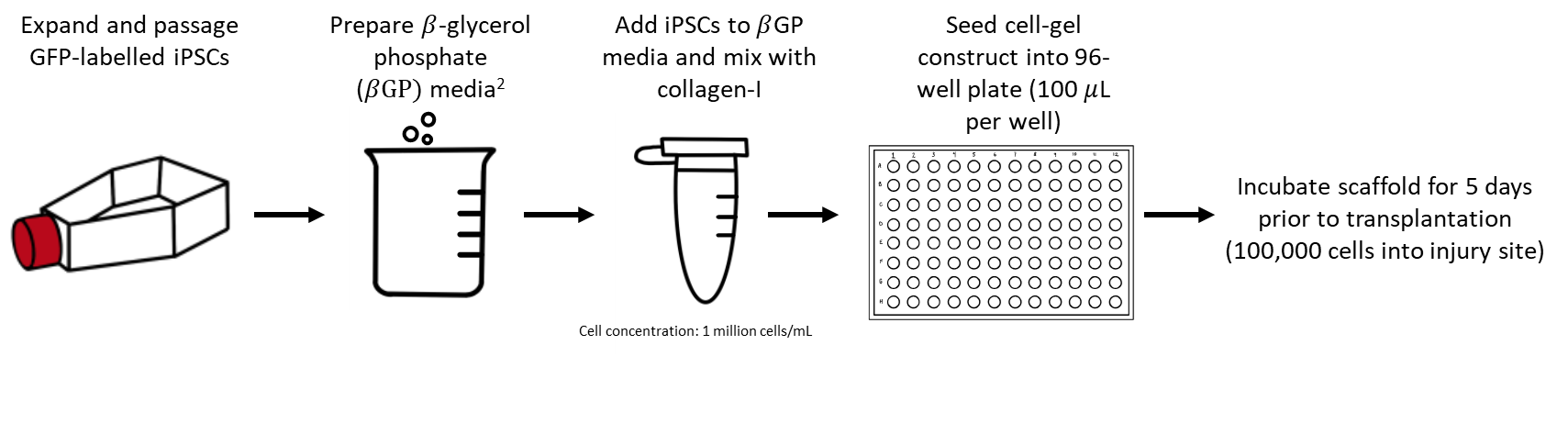


**Figure S1. Collagen gel preparation.** GFP-labelled iPSCs were expanded and then seeding in a Collagen-I scaffold. These scaffolds were polymerized with βGP at a final cell density of 100,000/construct.


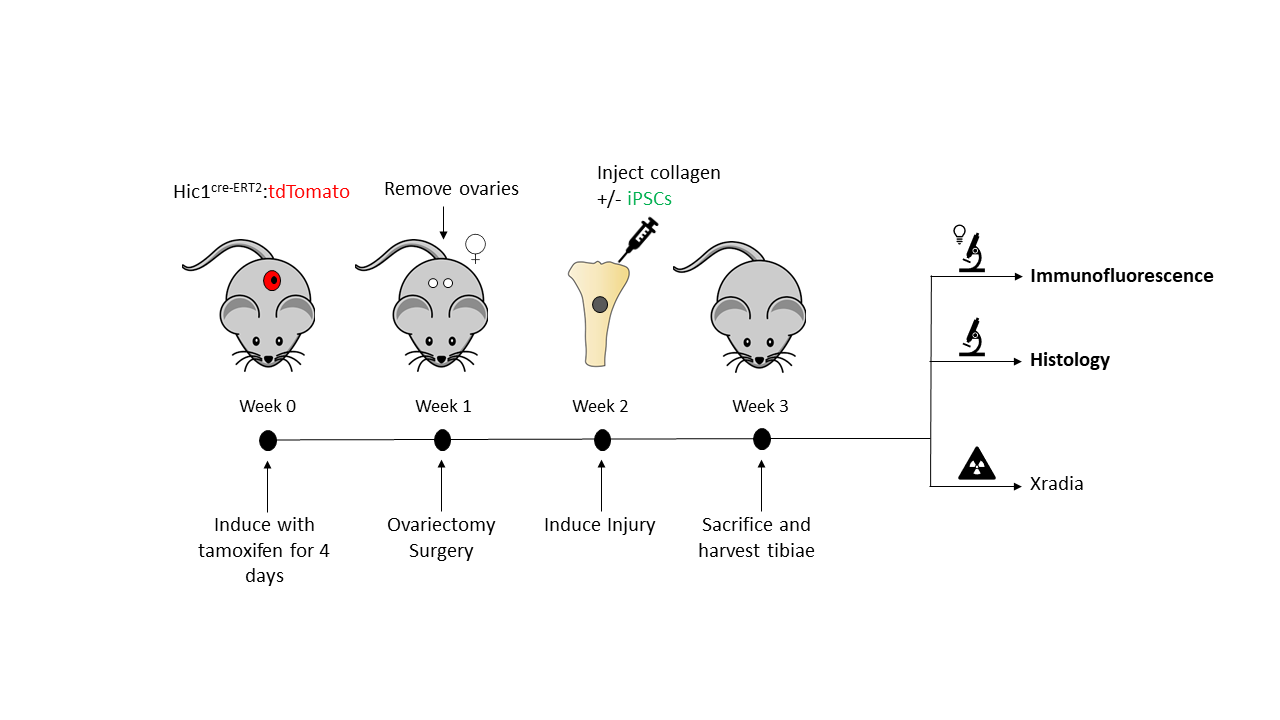


**Figure S2. Normal and OVX model experimental design.** One week post-tamoxifen induction, mice received OVX surgery. One week post-OVX, mice received the Burr-hole surgery. One week post-Burr-hole surgery, the mice were sacrificed and tibia were harvested for: histology/immunofluorescence; and Xradia imaging. Mouse image downloaded from Mouse Clipart Tumor - Mouse Clip Art. All other icons from Microsoft Office Icons.


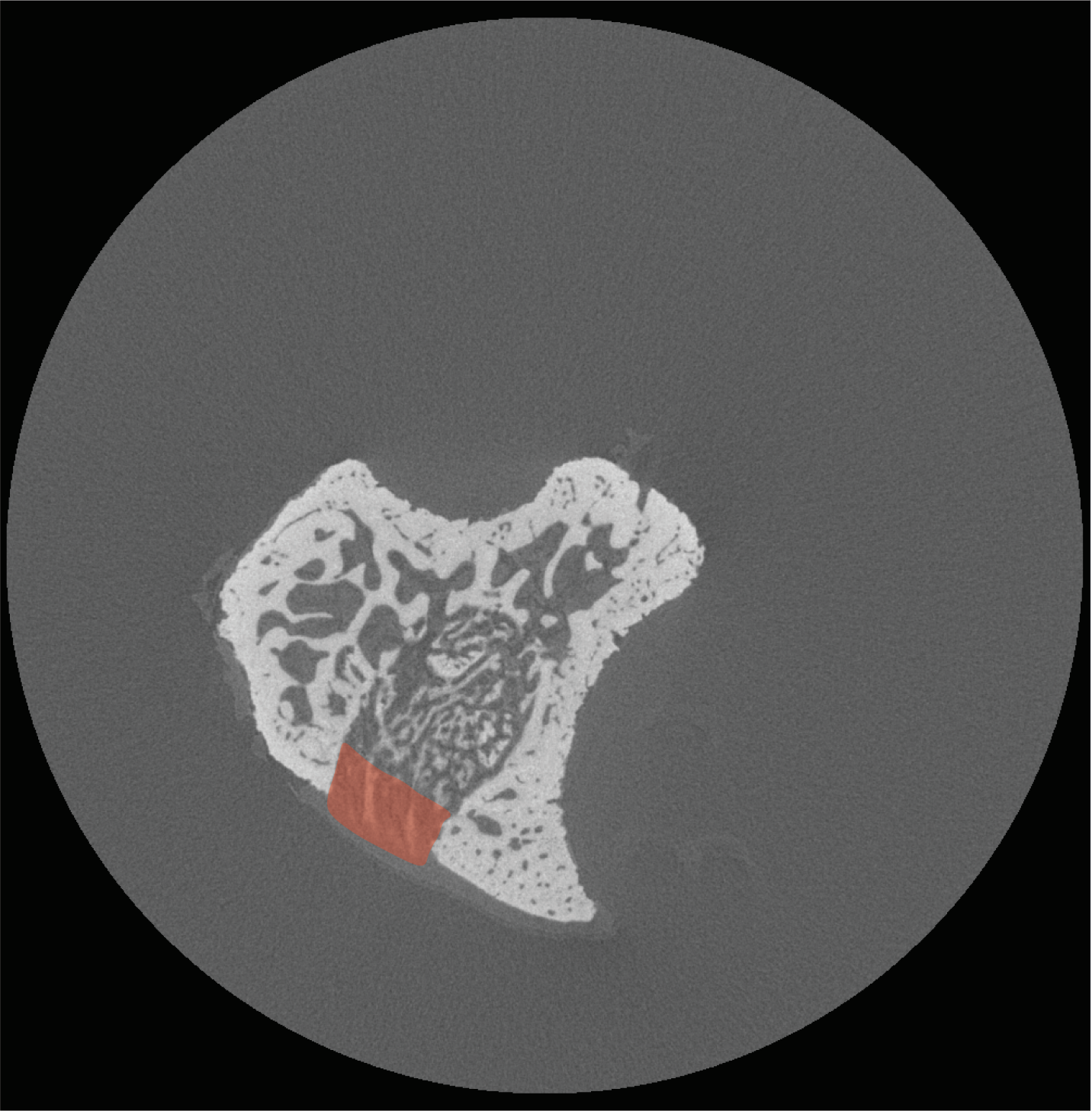


**Figure S3. Region of interest (highlighted in red) for XRM quantification.**


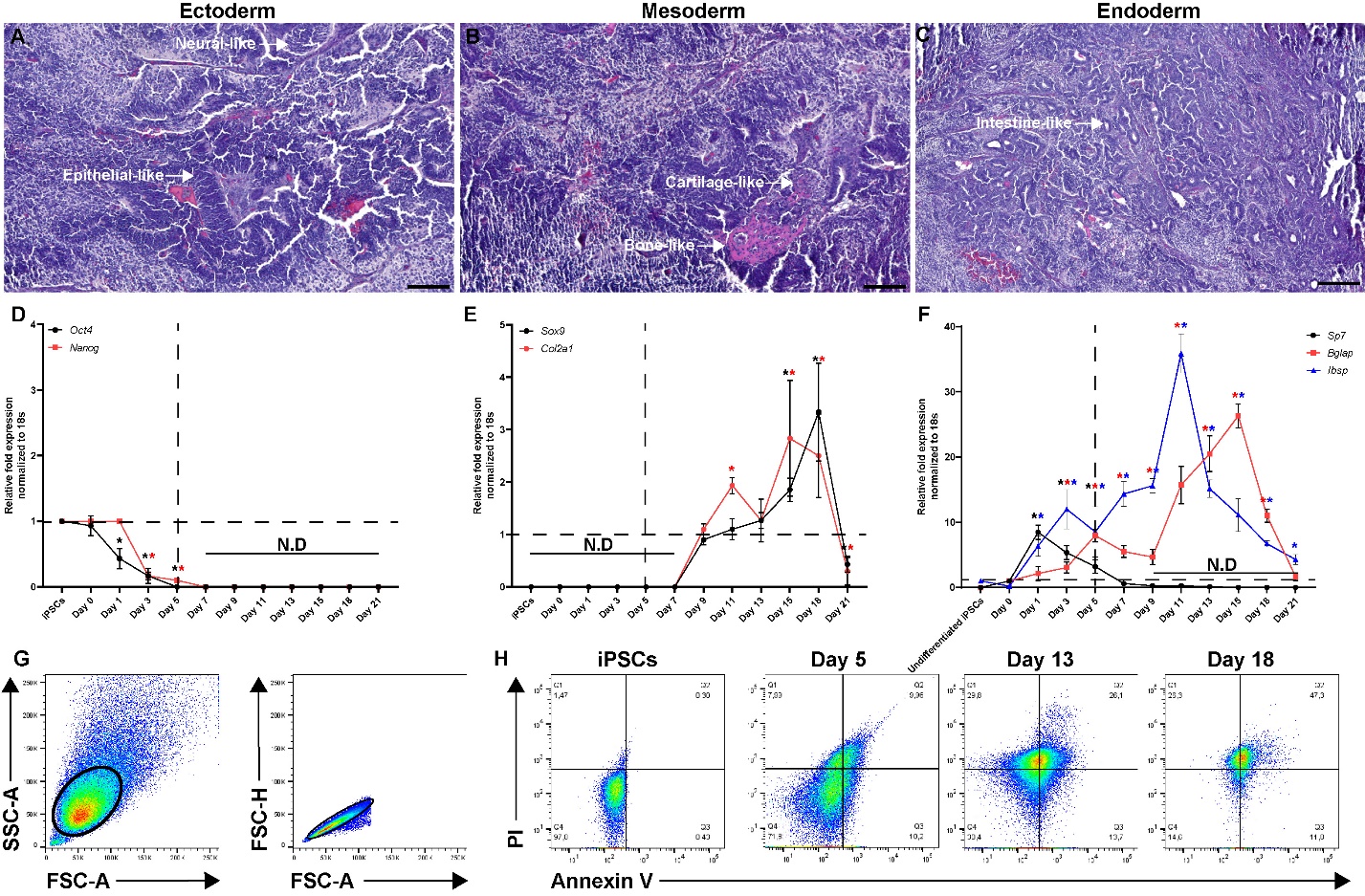


**Figure S4. Validation of pluripotency *in vivo* and osteogenic differentiation *in vitro*.** iPSCs were injected into SCID-Beige mice and the resultant teratomas were histologically assayed for ectoderm (A), mesoderm (B) and endoderm (C) -like tissues. iPSCs were loaded into collagen I scaffolds were incubated for 21 days *in vitro* and examined for the pluripotency markers (*Oct4* and *Nanog*) (D), chondrocyte markers (*Sox9, Col2a1*) and osteoblast markers (*Sp7*, *Bglap* and *Ibsp)*. Relative fold changes in expression compared to undifferentiated iPSCs and normalized to 18s are presented. N.D. = not detected. * *p*<0.05.


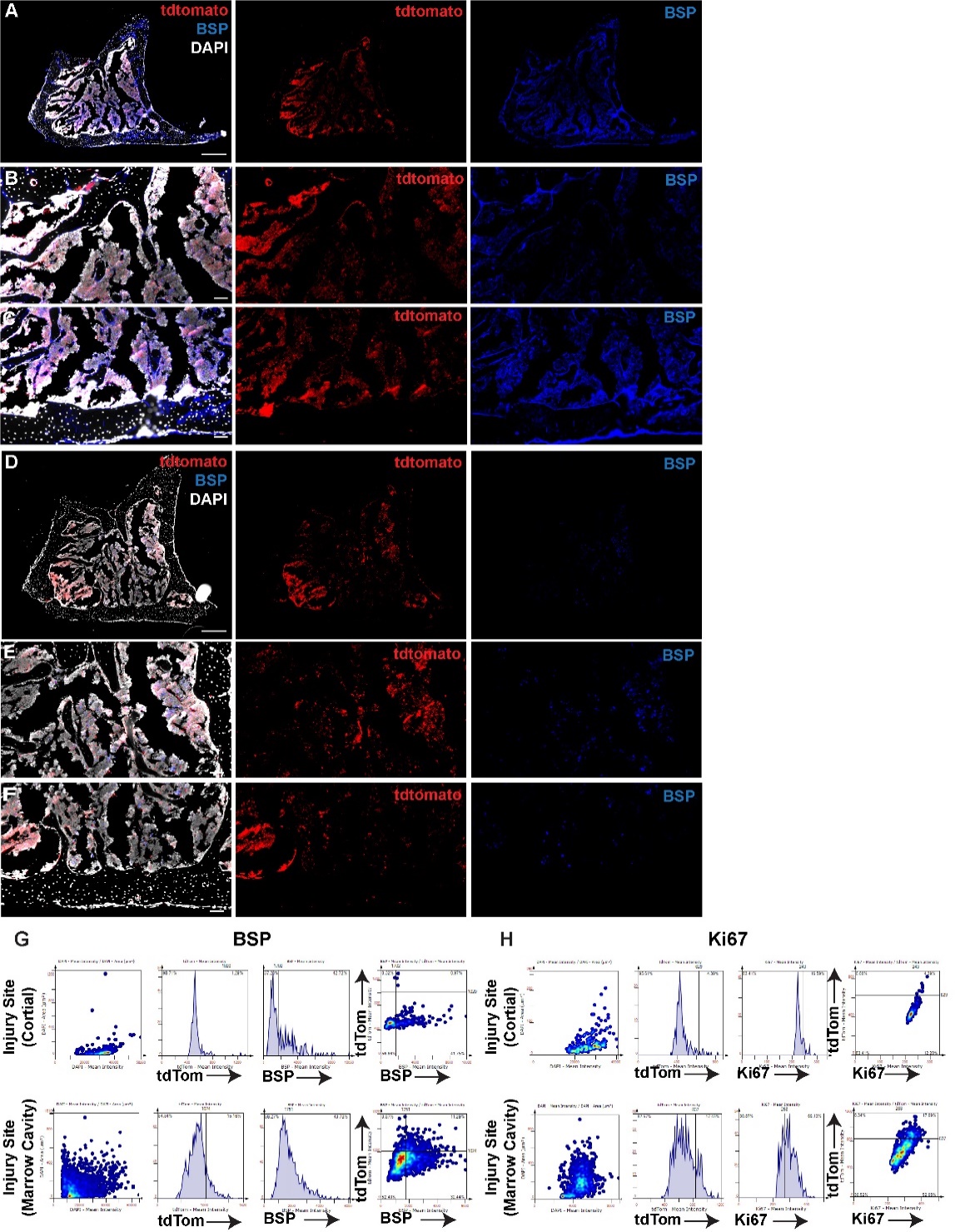


**Figure S5. Lineage tracing of MPCs in uninjured bone.** Uninjured bone from normal (A-F) mice were examined for the localization of *Hic1*^+^ lineage traced MPCs (tdTomato) and BSP (A-C) or Ki67 (D-F). Scale bars in A,D = 200µm, scale bars in B,C,E,F = 100µm. Representative tissue cytometry gates from the same groups (G,H).

*
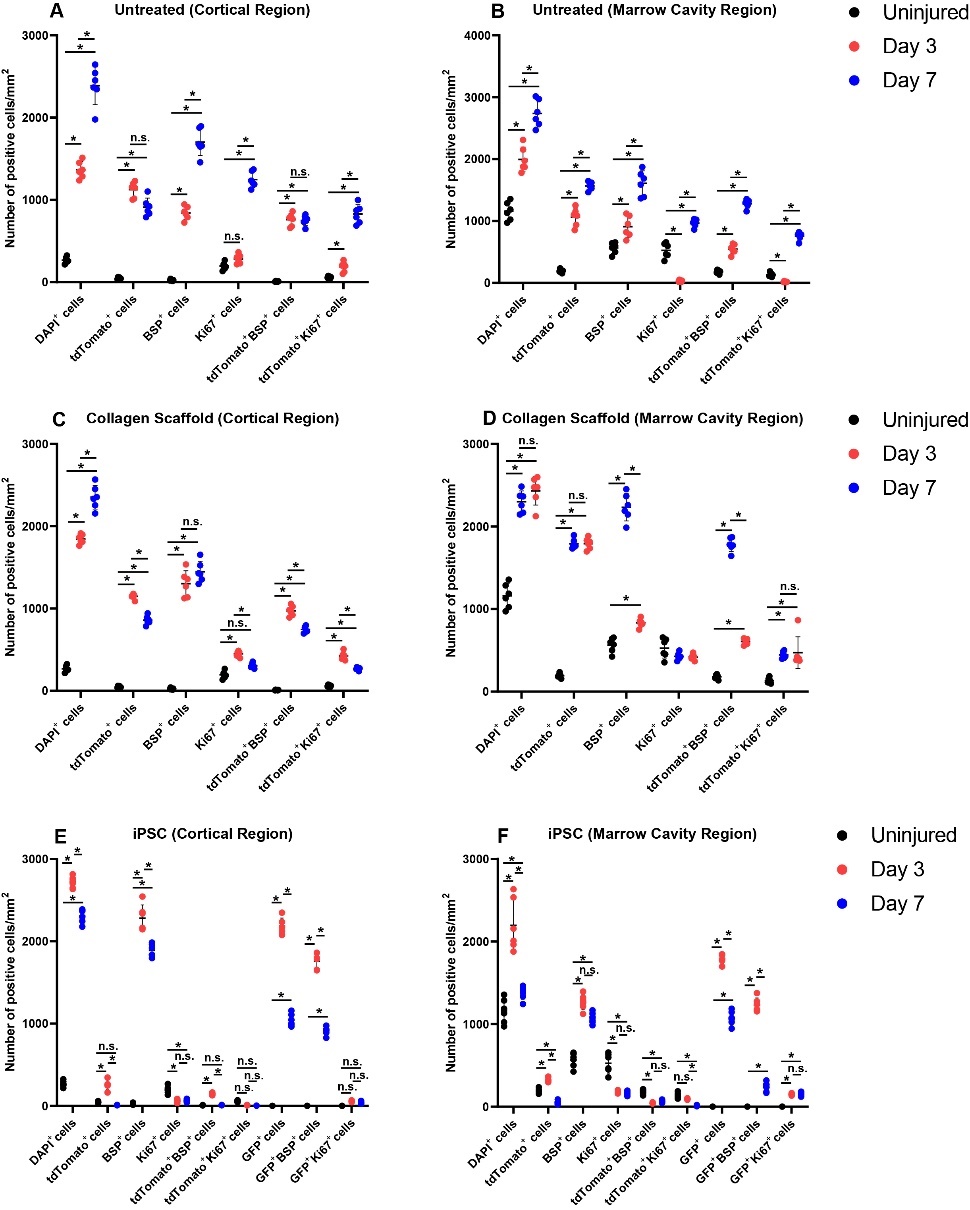
*

**Figure S6. Quantification of cell populations within the cortical and marrow compartments of the injury site.**  Tissue cytometry data was quantified, and the results examined statistically. The number of DAPI^+^, tdTomato^+^, BSP^+^, Ki67^+^, tdTomato^+^BSP^+^, tdTomato^+^Ki67, GFP^+^ iPSCs, GFP^+^Ki67^+^ GFP^+^BSP^+^ cell populations were quantified in untreated (A,B), collagen scaffold (C,D) and collagen scaffold plus iPSC (E,F) treatment groups. n.s. = not significant. *=p<0.05.


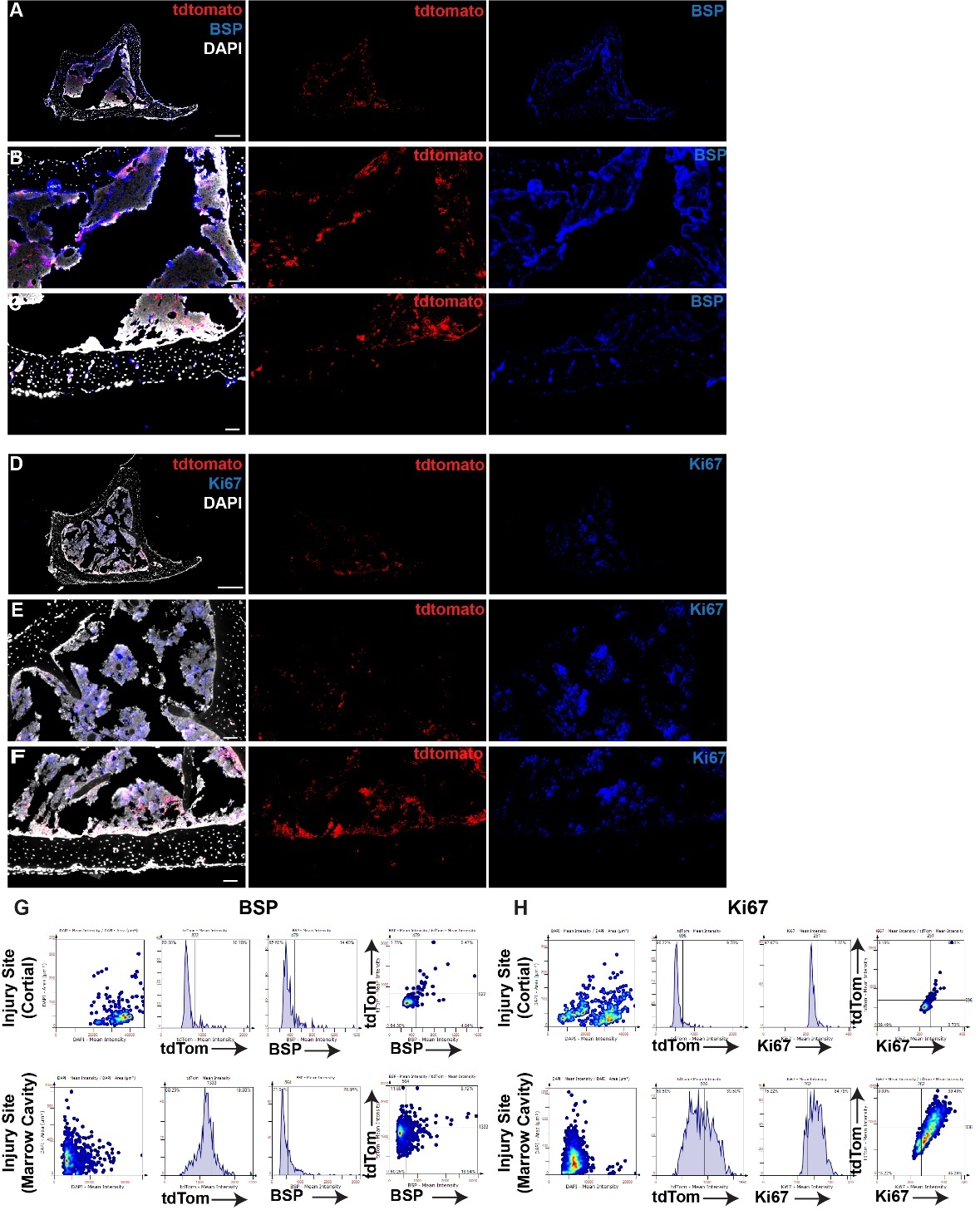


**Figure S7. Lineage tracing of MPCs in uninjured bone post-OVX.** Uninjured bone from OVX (A-F) mice were examined for the localization of *Hic1*^+^ lineage traced MPCs (tdTomato) and BSP (A-C) or Ki67 (D-F). Scale bars in A,D = 200µm, scale bars in B,C,E,F = 100µm. Representative tissue cytometry gates from the same groups (G,H).


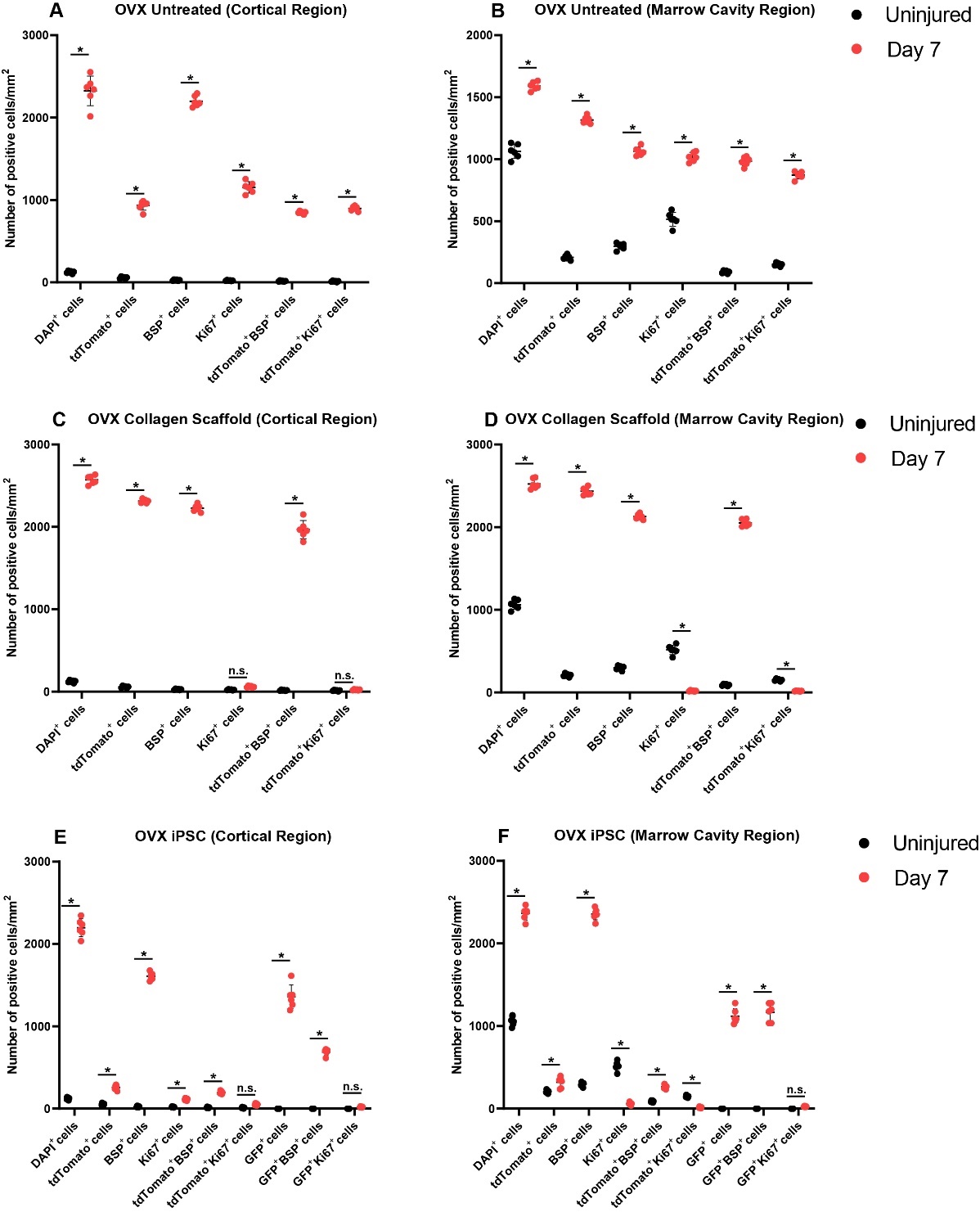


**Figure S8. Quantification of cell populations within the cortical and marrow compartments of the injury site in OVX mice.**  Tissue cytometry data was quantified, and the results examined statistically. The number of DAPI^+^, tdTomato^+^, BSP^+^, Ki67^+^, tdTomato^+^BSP^+^, tdTomato^+^Ki67, GFP^+^ iPSCs, GFP^+^Ki67^+^ GFP^+^BSP^+^ cell populations were quantified in untreated (A,B), collagen scaffold (C,D) and collagen scaffold plus iPSC (E,F) treatment groups. n.s. = not significant. *=p<0.05.


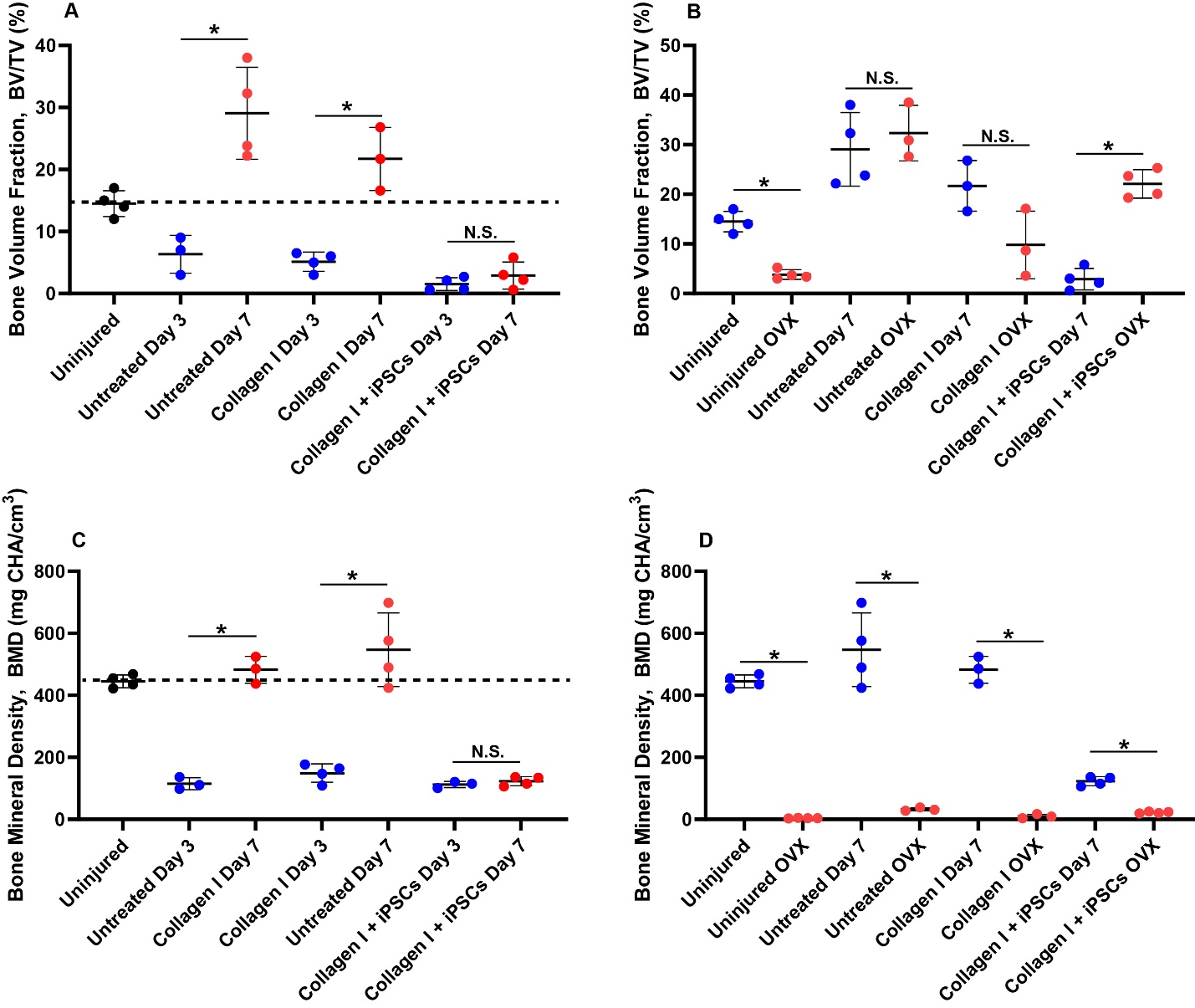


**Figure S9. Quantification of bone parameters within the site of intact vs OVX mice.** BV/TB (A,B), BMD (C,D) was quantified within the intact mice at 3 and 7 days post-injury (A,C) or intact vs. OVX mice at 7 days post-injury (B,D). n.s. = not significant. *=p<0.05.
